# Cohesin and Polycomb Proteins Functionally Interact to Control Transcription at Silenced and Active Genes

**DOI:** 10.1371/journal.pgen.1003560

**Published:** 2013-06-20

**Authors:** Cheri A. Schaaf, Ziva Misulovin, Maria Gause, Amanda Koenig, David W. Gohara, Audrey Watson, Dale Dorsett

**Affiliations:** Edward A. Doisy Department of Biochemistry and Molecular Biology, Saint Louis University School of Medicine, Saint Louis, Missouri, United States of America; Centre National de la Recherche Scientifique, France

## Abstract

Cohesin is crucial for proper chromosome segregation but also regulates gene transcription and organism development by poorly understood mechanisms. Using genome-wide assays in Drosophila developing wings and cultured cells, we find that cohesin functionally interacts with Polycomb group (PcG) silencing proteins at both silenced and active genes. Cohesin unexpectedly facilitates binding of Polycomb Repressive Complex 1 (PRC1) to many active genes, but their binding is mutually antagonistic at silenced genes. PRC1 depletion decreases phosphorylated RNA polymerase II and mRNA at many active genes but increases them at silenced genes. Depletion of cohesin reduces long-range interactions between Polycomb Response Elements in the *invected-engrailed* gene complex where it represses transcription. These studies reveal a previously unrecognized role for PRC1 in facilitating productive gene transcription and provide new insights into how cohesin and PRC1 control development.

## Author Summary

An important task for the cohesin protein complex that binds chromosomes is to ensure equal distribution of chromosomes into the daughter cells when a cell divides. Small changes in cohesin activity, however, can alter gene activity without affecting chromosome distribution, and disrupt physical and mental development. How cohesin controls gene activity and development is not well understood. In this study we show that cohesin controls the binding of the Polycomb Repressive Complex 1 (PRC1) to many genes. PRC1 silences many genes that control development. Surprisingly, we find that cohesin aids binding of PRC1 to active genes, where PRC1 ensures that RNA polymerase, the enzyme that transcribes genes, is properly modified before entering the gene body. We also find that cohesin antagonizes the binding and activity of PRC1 at genes silenced by PRC1, and can influence interactions between the DNA sequences that recruit PRC1 and other Polycomb complexes to silenced genes. These findings provide new and unexpected insights into how both cohesin and PRC1 control gene activity during development.

## Introduction

The cohesin complex that encircles DNA is named for its role in mediating sister chromatid cohesion [1]. Cohesin has four subunits: the Smc1 and Smc3 *s*tructural *m*aintenance of *c*hromosomes proteins, Rad21, a kleisin protein, and Stromalin (SA). Cohesin is loaded onto chromosomes by the kollerin complex containing the Nipped-B (NIPBL, Scc2) and Mau-2 (Scc4) proteins, and removed by the releasin complex containing Pds5 and Wapl.

Minor alterations in cohesin function disrupt development without affecting sister chromatid cohesion or chromosome segregation [2]. In humans, dominant loss-of-function mutations in the *NIPBL* gene encoding a kollerin subunit, and dominant missense mutations in the Smc1 or Smc3 cohesin subunits cause Cornelia de Lange syndrome (CdLS) [3]. CdLS is characterized by poor growth, diverse structural abnormalities and intellectual impairment. Current data argue that small changes in cohesin function disrupt development because cohesin binds and regulates many genes important for growth and development [2], .

Genetic and molecular evidence suggests that cohesin also functionally interacts with Polycomb group (PcG) epigenetic silencing proteins during development. There are two major PcG protein complexes: Polycomb Repressive Complex 2 (PRC2), whose Enhancer of zeste [E(z)] subunit performs histone 3 lysine 27 trimethylation (H3K27me3), and PRC1, whose Polycomb (Pc) subunit binds the H3K27me3 histone modification [5]–[8]. The Drosophila Rad21 cohesin subunit is encoded by *verthandi* (*vtd*). *Nipped-B* kollerin and *vtd* cohesin subunit mutations suppress the body segment transformations of *Pc* mutant flies, and a *wapl* releasin mutation that stabilizes cohesin binding causes phenotypes similar to *Pc* mutations [9]–[11].

The *in vivo* findings argue that cohesin antagonizes PcG silencing at certain genes, and consistent with this idea, genome-wide mapping in Drosophila cells revealed that cohesin and Nipped-B preferentially bind promoters of active genes, and are usually excluded from PcG-silenced genes [12]. The few exceptional genes that bind cohesin and also have the PRC2-generated H3K27me3 mark are not fully silenced, and show unusually large increases in expression upon cohesin depletion [13]. Cohesin biochemically interacts with PRC1, suggesting that these two complexes might directly control each other's activities [14].

To clarify the functional relationships between cohesin and PcG complexes, we mapped their binding genome-wide in developing Drosophila wing and cultured nervous system cells, and measured the effects of cohesin on PRC1 binding, and *vice versa*. We also measured the genome-wide effects of PRC1 depletion on Pol II occupancy and mRNA levels. These studies revealed functional interactions between cohesin and PRC1 that give rise to their co-regulation of active genes, and antagonism to each other on expression of PcG-silenced genes. They uncovered an unexpected role for PRC1 in controlling RNA polymerase II (Pol II) function at active genes that provides new insights into how cohesin and PcG proteins regulate transcription.

## Results

### Cohesin and PRC1 Bind Active Genes in the Wing Imaginal Disc

The genomic data from cultured cells and genetic interactions between cohesin and PcG mutations suggest that cohesin and PcG proteins functionally interact to control gene expression and development. To test this idea in a developing tissue, we used genomic chromatin immunoprecipitation (ChIP-chip) to map Rad21, Nipped-B, RNA polymerase II (Pol II), and PRC2-generated histone 3 lysine 27 trimethylation (H3K27me3) in late 3^rd^ instar Drosophila wing imaginal discs. Genetic data shows that both cohesin and PcG proteins regulate gene expression and development in wing discs (e.g. [15]–[17]).

Two biological replicates were used for each ChIP-chip experiment, and enrichment was calculated genome-wide using the model-based MAT algorithm [18], [19]. We found that the cohesin binding patterns seen in Drosophila cell lines [12] hold true in developing wing: (a) cohesin (Rad21) co-localizes with Nipped-B (genome-wide correlation r = 0.91), (b) cohesin and Nipped-B preferentially bind active genes occupied by Pol II (r = 0.63), and (c) cohesin and Nipped-B are largely absent from silenced genes with PRC2-generated H3K27me3 (r = 0.24). The genome-wide correlation coefficients are summarized in Table S1. These and the other genome-wide ChIP and expression assays performed for this study (see below) are listed in Table S2. Table S3 lists all annotated genes, and indicates whether or not they bind each of the factors that were mapped by ChIP-chip in wing discs and cultured cells (see below). Table S3 also gives the mRNA expression values determined by microarray.

The correlation between cohesin and H3K27me3 is higher in wing discs than in cultured cells. It is likely that sites of false overlap arise from the mixture of different cell types in the wing disc, in which a gene can be silent in one compartment and active in another. For example, the *invected-engrailed* (*inv-en*) gene complex, encoding two homeobox transcription factors that confer posterior fate, is expressed in the posterior wing compartment and PcG-silenced in the anterior (e.g. [16], [20]).

**Figure 1:**
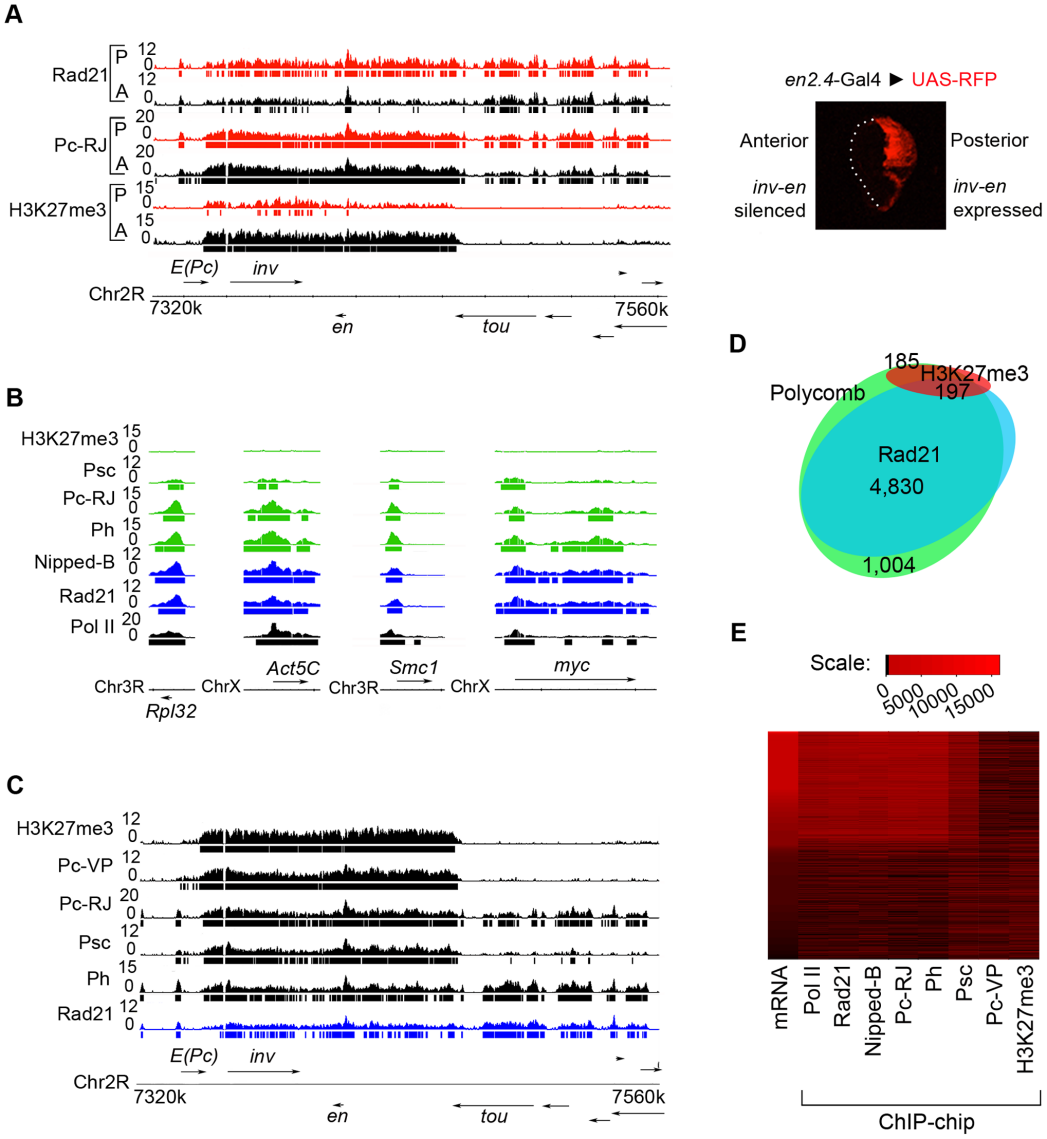
PRC1 binds both silenced genes and active cohesin-binding genes in wing imaginal discs. (**A**) ChIP-chip tracks for Rad21 (cohesin), Polycomb (PRC1; Pc-RJ), and H3K27me3 (PRC2) of the *invected-engrailed* gene complex in anterior (A, black) and posterior (P, red) wing discs. Bars underneath ChIP tracks indicate significance at p≤10^−3^. A wing disc expressing red fluorescent protein (RFP) under control of *en2.4*-GAL4 is shown to the right. (**B**) RNA polymerase II (Pol II, black, 8WG16 antibody), Rad21 and Nipped-B (blue), PRC1 subunits (Psc, Pc, Ph) and PRC2-generated H3K27me3 (green) ChIP at constitutively active genes in whole wing discs. A higher resolution view of the *dm/myc* gene is in Figure S4. (**C**) Rad21, PRC1 (Ph, Psc, Pc-VP, Pc-RJ) and H3K27me3 ChIP at *invected-engrailed* using whole wing discs. (**D**) Venn diagram of the overlap of Rad21 (cohesin), Polycomb (Pc, PRC1), and H3K27me3 (PRC2) binding to all annotated genes in posterior wing imaginal discs. Binding was called at threshold of p≤10^−3^. There are 4,830 genes that bind both Rad21 and Pc, 1,004 genes that bind Pc but not Rad21, 185 genes that bind Pc and H3K27me3, and 187 genes that bind Pc, H3K27me3 and Rad21. In the latter group, only a few such as *invected* and *engrailed* show the large overlapping domains of cohesin and H3K27me3 extending over several kilobases that we define as the cohesin-H3K27me3 restrained state [13]. (**E**) Association of PRC1 subunits with active genes in whole wing discs. The mRNA levels for 13,132 genes measured by microarray are sorted from lowest to highest according the heat map. The aligned heat maps show the total ChIP signal (Figure S7, method 1) for the indicated proteins for each gene.

The *inv-en* complex is of particular interest because it is one of the rare examples of cohesin-H3K27me3 overlap in BG3 cells, where its expression is highly sensitive to cohesin dosage [13]. We thus mapped cohesin (Rad21), H3K27me3, and the PRC1 subunit Polycomb (Pc) in the posterior and anterior wing disc separately to determine if *inv-en* might be regulated by both cohesin and PcG proteins in either the active or silenced state. We used a transgene that expresses red fluorescent protein (RFP) only in the posterior compartment [21] as a guide to slice the discs into anterior and posterior portions (Figure 1A).

We found that *inv-en* binds cohesin primarily in the posterior disc, where it is expressed, and is marked by H3K27me3 primarily in the anterior disc, where it is silenced (Figure 1A). The low levels of cohesin detected in anterior chromatin, and low levels of H3K27me3 seen in posterior chromatin, likely reflect imperfect dissection (10–20% cross-contamination by visual inspection and by measuring *inv* and *en* RNA levels, Figure S1). This low level of contamination is unavoidable, given the difficulty of the dissection by hand of discs that are some 250 by 350 microns in size. Tissue dissociation and FACS sorting did not provide sufficient viable cells for chromatin preparation. Nonetheless, the data clearly demonstrate an inverse relationship between H3K27me3 and cohesin at the *inv-en* complex between the anterior and posterior compartments. The genome-wide correlation between Rad21 and H3K27me3 in the posterior wing disc (r = 0.11) is significantly lower than for whole discs (r = 0.24), further confirming that some sites of Rad21-H3K27me3 overlap seen with whole discs reflect tissue heterogeneity, and that H3K27me3 and cohesin have an inverse relationship genome-wide in wing discs as in cultured cells (Table S1).

We unexpectedly found that the Pc PRC1 subunit occupies *inv-en* and flanking active genes at virtually equal levels in anterior and posterior wing discs (Figure 1A). Even more surprising, by measuring the mRNA levels for some 13,000 genes in whole wing discs (Table S3) and comparing to the genomic ChIP data, we found that Pc associates with a large portion of active genes, including ubiquitously expressed genes such as the *Act5C* actin gene, *diminutive* (*dm*, the Drosophila *myc* gene) and some ribosomal protein genes (Figure 1B). Among active genes, Pc preferentially occupies those that bind cohesin (Figure 1D). In the posterior disc, Pc and Rad21 binding exhibit a genome-wide correlation of 0.75 (Table S1), and Pc is present at 90% of cohesin-bound genes (Figure 1D). Conversely, 76% of Pc sites exhibit cohesin binding.

The extensive overlap of cohesin and Pc at active genes was unexpected, given that PRC1 is generally thought to associate primarily with PcG-silenced genes. For instance, Pc was previously mapped in Drosophila cell lines with a different antibody (denoted Pc-VP; [22]) than the one used here (denoted Pc-RJ; [23]), and was generally found at silent genes marked by H3K27me3 [24], [25]. Both Pc-VP and Pc-RJ were made using the same Pc fragment as antigen (residues 191–354) and antigen-affinity purified. The Pc-VP antibody was validated for ChIP-chip in the initial studies, and re-validated by the modENCODE project, including RNAi depletion experiments. Two laboratories independently verified the Pc-RJ antibody by RNAi, western blots and ChIP [13], [23]. Figure S2 confirms that the Pc-RJ antibody detects a single protein of the expected size that is reduced in cultured cells subjected to Pc RNAi treatment.

The core PRC1 complex consists of Pc, Polyhomeotic (Ph), Posterior sex combs (Psc) and Sex combs extra (Sce/dRing). We thus further mapped PRC1 in whole wing discs using the Pc-VP Pc antibody and validated antibodies against the Polyhomeotic (Ph) and Posterior sex combs (Psc) PRC1 core subunits [22]. These antibodies were previously validated by westerns, *in vivo* expression of fusion proteins, and ChIP-chip [22], [24]–[26]. Figure S2 shows that the Ph antibody recognizes one major band of the correct size that is reduced upon treatment of cultured cells with Ph RNAi.

These experiments demonstrate that the PRC1 complex is present at active cohesin-binding genes in wing discs. Ph ChIP gave a nearly identical pattern to Pc-RJ, with a genome-wide correlation with Rad21 of 0.82 in whole discs (Figure 1B,C,E; Table S1; Figure S3). Thus two validated antibodies against different PRC1 subunits show essentially the same pattern. It is very unlikely that both cross-react with a non-PRC1 protein that co-localizes with cohesin by chance. Moreover, Psc was also detected at many cohesin-binding active genes, although the signals are noticeably lower than at silenced genes (Figure 1B,C,E; Figure S3). We cannot distinguish if the lower Psc signals reflect reduced epitope accessibility, or if Psc is present in a smaller fraction of the PRC1 complexes at active genes. For instance, Su(z)2, a Psc homolog encoded by a neighboring gene, may substitute for Psc in a much higher fraction of PRC1 complexes at active genes.

Figure S3 shows plots of the ChIP enrichment at all microarray features for each of the PRC1 subunits against each other over a 400 kb region on chromosome 2L that includes several active genes and the PcG-silenced *dpp* gene, along with the genome-wide correlation coefficient for each comparison. Ph shows a high genome-wide correlation with Pc-RJ (0.85), while Pc-VP shows a lower correlation, with the plots showing a clear separation of active and silenced genes. Psc correlates with Ph at both silenced and active genes, with a distinct separation into silenced genes with high Psc and active genes with low Psc. Figure S4 shows a high resolution view of the *diminutive* (*dm*, *myc*) gene to show the close similarities in the ChIP patterns of three PRC1 subunits (Pc, Ph, Psc) at a constitutively-active cohesin-binding gene. Figure S5 shows that additional independent anti-Pc and anti-Ph antibodies [27]–[29] also detect PRC1 at active cohesin-binding genes by ChIP-qPCR. We thus conclude that PRC1 is present at most active cohesin-binding genes.

As in cultured cells, the Pc-VP antibody detected Pc binding primarily at sites of H3K27me3 in wing discs, such as *inv-en* (Figure 1C,E). We do not know why Pc-VP, in contrast to several other PRC1 antibodies, does not detect PRC1 at active genes, but one clear possibility is that the Pc epitope recognized by Pc-VP is masked at active cohesin-bound loci. This idea is consistent with the direct interaction between cohesin and PRC1 detected by purification of biotin-tagged PRC1 [14]. The cohesin-PRC1 interaction was characterized using different cohesin and PRC1 antibodies than those used in our studies. With the antibodies we used for genomic ChIP, immunoprecipitation of Rad21 from soluble nuclear extracts treated with DNase I co-precipitated Pc and Ph, confirming that cohesin and PRC1 interact (Figure S2). These results also provide further validation of the specificity of the Pc-RJ and Ph antibodies.

### Cohesin Facilitates PRC1 Binding to Active Genes

The extensive overlap of cohesin and PRC1 at active genes in wing discs and the direct interaction between cohesin and PRC1 suggested that they might influence each other's binding. The presence of multiple cell types in wing discs creates ambiguities in interpreting ChIP signals at genes that are not active or silent in all cells. It is also difficult to reduce cohesin and PRC1 levels *in vivo* by more than 50% without causing lethality or substantially altering development. Homozygous cohesin and PcG mutants are early lethals, and many putatively tissue-specific RNAi drivers cause lethality even without large reductions in cohesin [30]. We thus chose to examine the effects of cohesin on PRC1 binding and *vice versa* in cultured ML-DmBG3 (BG3) cells derived from 3^rd^ instar central nervous system as a more homogenous cell population, and in which cohesin or PRC1 subunits can be easily reduced by at least 80% using RNAi without causing lethality [13].

**Figure 2:**
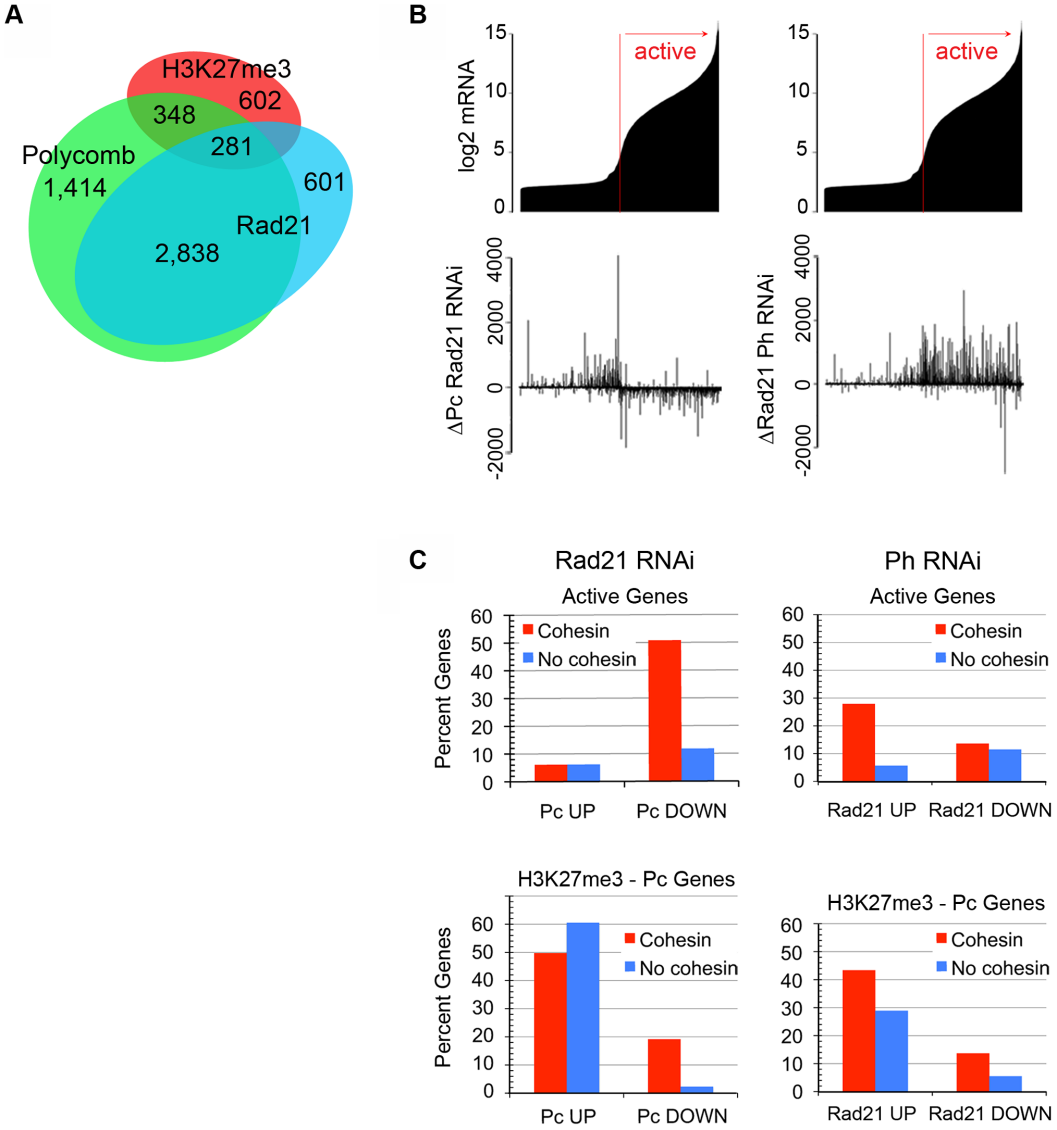
Cohesin and PRC1 affect each other's binding to active and PcG-silenced genes in cultured BG3 cells. (**A**) Overlap of Rad21 (cohesin), Polycomb (Pc, PRC1) and H3K27me3 (PRC2) binding at all annotated genes. Binding was called at a statistical threshold of p≤10^−2^. (**B**) The top graphs show the mRNA levels (log2 scale) for 13,132 genes measured by microarray in control BG3 cells. The aligned bar plots just below show the change in total Pc ChIP signal for each gene after Rad21 depletion and change in total Rad21 ChIP signal after Ph depletion (Figure S7, method 1). (**C**) Percent of cohesin-binding (red) and non-binding genes (blue) that show an increase (UP) or decrease (DOWN) in Pc ChIP signal after Rad21 depletion, and genes that show an increase or decrease in Rad21 after Ph depletion. The genes are subdivided into active genes (∼7,000), and the genes marked by H3K27me3 that bind Pc (∼600 genes because not all inactive genes are PcG-silenced). Cohesin binding was called at a statistical threshold of p≤10^−3^ and increases and decreases were called by differences in ChIP signal that were at least 2 standard deviations from the median genome-wide difference that extend for at least 105 bp (Figure S7, method 2).

**Figure 3:**
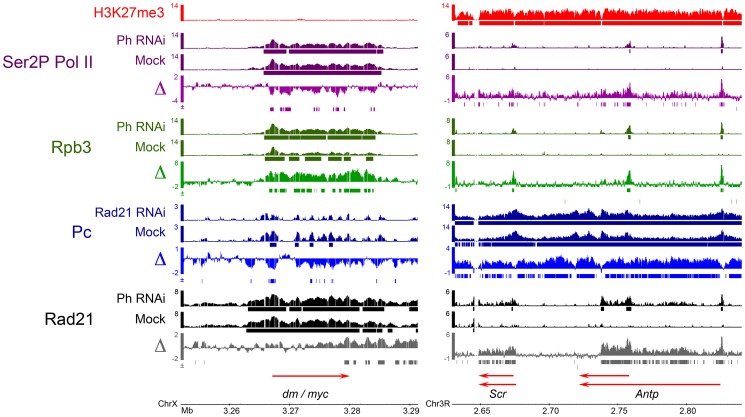
Examples of effects of cohesin and PRC1 on each other's binding, and effects of PRC1 depletion on Pol II occupancy in BG3 cells. ChIP-chip tracks of Rad21, Polycomb (Pc, PRC1), Rpb3 (total Pol II), or Ser2P Pol II (elongating Pol II), from control (mock-treated) BG3 cells and Rad21 or Polyhomeotic (Ph, PRC1) depleted cells are shown. Bars underneath the ChIP tracks indicate binding at the p≤10^−3^ threshold. The Δ tracks show the differences in the ChIP enrichment (MAT score) between the RNAi depleted and control cells. Bars underneath the Δ tracks indicate significant increases (+) or decreases (−) in ChIP enrichment after RNAi treatment, using method 2 in Figure S7. The *myc* (*diminutive*, *dm*) gene, is an example of an active, cohesin-PRC1 binding gene. *Sex combs reduced* (*Scr*) and *Antennapedia* (*Antp*) in the Antennapedia complex (ANT-C) are examples of PcG-silenced genes.

We mapped Pc and Rad21 binding genome-wide in BG3 cells using an antibody (Pc-RJ) that detects PRC1 at active genes. Cohesin binds primarily at active genes with promoter-proximal paused RNA polymerase II (Pol II) in BG3 cells, and controls the transition of paused Pol II to elongation [12], [13], [31], [32]. There is extensive overlap between cohesin and Pc in BG3 cells, although lower than seen in wing discs, with 72% of cohesin-bound genes binding Pc (Figure 2A). We conclude, therefore, that PRC1 also binds most active cohesin-binding genes in BG3 cells, including several highly-expressed constitutively-active genes such as some ribosomal protein genes, *Act5C*, and *dm/myc* (Figure 3).

We tested if cohesin and PRC1 influence each other's binding by performing genomic ChIP of Rad21 after Ph RNAi depletion, and Pc ChIP after Rad21 RNAi depletion, with two biological replicates for each RNAi treatment. Depletion of cohesin by 80% in BG3 cells does not alter chromosome segregation, cell morphology or viability, and modestly decreases proliferation [13], [31]. This depletion reduces cohesin-binding by 70% or more at genes with high levels of cohesin, and large changes in expression of several genes that bind cohesin [13],[31],[32]. Figure S6 shows that cohesin depletion increases the fraction of cells in G2 without a substantial effect on the proportion of cells in S phase. Prior analysis, including genome-wide mRNA measurements, suggests that the G2 delay reflects decreased *diminutive* (*dm*, *myc*) gene expression and cell differentiation, and not mitotic defects [13]. There are minimal effects on sister chromatid cohesion, and no detectable effects on chromosome segregation. There is a modest 1.5-fold increase in *cyclin B* mRNA and modest decrease in expression of genes involved in spindle formation and elongation, indicating a delay prior to entry into mitosis. There are no changes in expression of cell cycle checkpoint or apoptosis genes. Consistent with decreased *dm/myc* expression, the most significant down-regulated gene ontology is protein synthesis, and the top up-regulated gene ontology is development, suggesting a change in cell differentiation. Both the up- and down-regulated genes are highly enriched for cohesin-binding genes, indicating that a large proportion of the affected genes are directly regulated by cohesin, and that most expression changes are not attributable to changes in cell physiology. Although the levels of mRNA produced by most genes that do not bind cohesin do not change upon cohesin depletion, direct transcription measurements show that transcription initiation subtly decreases at most of them, which likely reflects decreased levels of Dm/Myc or other general transcription factors [32].

Similar to cohesin depletion, PRC1 depletion also increases the fraction of cells in G2 without decreasing the proportion of S phase cells (Figure S6). Depletion of Ph, similar to Pc depletion [13] does not reduce viability, but causes BG3 cells to form more distinct colonies, with longer cellular processes, and cessation of proliferation after six to seven days of treatment. We thus examined the effects of Ph on Rad21 binding after five days of RNAi treatment, when Ph is reduced by at least 80% (Figure S2) and cells are still dividing, to minimize binding changes that might be caused by cell differentiation.

Changes in Pc binding upon Rad21 depletion were measured by two methods (Figure S7), both of which showed that Rad21 depletion reduces Pc binding to active genes. In the first method, we integrated the Pc ChIP MAT scores for all microarray features within a gene body in the control and Rad21-depleted cells, and then calculated the difference in the total ChIP signal for each gene between the depleted and control cells (Figure S7, method 1). By comparing these differences to the control mRNA level for each gene (Table S3), we found that Pc binding decreases at many active genes upon Rad21 depletion (Figure 2B). We also measured the change in Pc ChIP enrichment after Rad21 depletion at each individual microarray feature (ΔPc) across the genome, and then mapped all locations where ΔPc was greater or smaller than the median genome-wide ΔPc by at least two standard deviations for at least three microarray features in a row against all annotated genes (Figure S7, method 2). These intervals are shown in the Δ tracks in Figure 3 and Figure S8. We found that 50% of active cohesin-binding genes show a Pc decrease, compared to only 10% of the genes that don't bind cohesin, and that Pc increases are 10-fold less frequent than increases at cohesin-binding genes (Figure 2C).

The finding that cohesin depletion frequently decreases but rarely increases PRC1 binding to active cohesin-binding genes argues that most of the changes in PRC1 binding are a direct consequence of cohesin reduction at the cohesin-binding gene, and not caused indirectly by cellular differentiation. Differentiation would be expected to have more selective, gene-specific effects, and would also be more likely to increase PRC1 at some active genes. Together with the direct interaction between cohesin and PRC1 (Strübbe et al., 2011; Figure S2), the decrease in PRC1 levels at many cohesin-binding genes argues that cohesin recruits or stabilizes PRC1 binding at active genes. Because cohesin depletion alters transcription of many genes, we cannot rule out the possibility that the PRC1 decreases upon cohesin depletion are caused in part by transcriptional changes. We note, however, that the effects of cohesin reduction on PRC1 binding do not fully mirror the effects on Pol II occupancy. Although cohesin depletion frequently reduces both total Pol II (Rpb3) and Pc at cohesin-binding genes, the genome-wide correlation between the change in Pc (ΔPc) and change in Rpb3 (ΔRpb3; [32]) is modest (r = 0.29).

In addition to the effects of cohesin depletion on PRC1 binding, we found that PRC1 depletion affected cohesin binding at active genes. Ph depletion increased Rad21 signals at nearly a third of active cohesin-binding genes (Figure 2B, Figure 2C, Figure 3, Figure S8). Cohesin increases were twice as frequent as decreases (Figure 2C), and increases were generally greater in magnitude than decreases (Figure 2B). These findings suggest that although cohesin facilitates PRC1 binding, PRC1 negatively influences cohesin binding at many active genes. We cannot rule out, however, the possibility that some increases in cohesin signals are caused by greater epitope accessibility in the absence of PRC1, or increased Pol II levels in the gene body (see below).

### Cohesin and PRC1 Binding Are Antagonistic at PcG-Silenced Genes

In contrast to active genes, cohesin and PRC1 binding are mutually antagonistic at PcG-silenced genes that are marked by H3K27me3 and bind Pc. Rad21 binding increases at 28% of these genes upon Ph depletion, and Pc binding increases at 60% of them upon Rad21 depletion (Figure 2B,C). The Rad21 increases upon Ph depletion, and the Pc increases upon Rad21 depletion occurred at all the genes in the Antennapedia and bithorax HOX gene complexes (ANT-C, BX-C). The changes at the *Sex combs reduced* (*Scr*) and *Antennapedia* (*Antp*) genes in the ANT-C are shown in Figure 3.

The increased cohesin binding is not caused by higher cohesin expression, because Ph depletion slightly reduces cohesin subunit mRNA levels, as determined from a genome-wide mRNA analysis described below. The increase in Rad21 at silenced genes, however, correlates with an increase in Pol II and mRNA for the silenced genes (see below), and thus increased cohesin binding to silenced genes could be caused by increased transcription. The increased Pc levels at silenced genes upon Rad21 depletion are unlikely to reflect higher PRC1 expression. Rad21 depletion increases the *Psc* and *Su(z)2* mRNAs less than 2-fold, and has no significant effect on the expression of other PRC1 subunits [13]. Also, as described above, Rad21 depletion decreases PRC1 levels at active genes. The increase in Pc at silenced genes upon Rad21 depletion does not reflect a change in the expression of the silenced genes, because they remain silenced.

Increases in Pc and Rad21 binding at silenced genes upon cohesin and PRC1 depletion were substantially more frequent than decreases. Pc increases were detected at 60% of the H3K27me3-Pc genes upon Rad21 depletion, but decreases were detected at only 2% (Figure 2C). At the H3K27me3-Pc genes that bind cohesin, some of which are not fully silenced, Pc increases were 2.5-fold more frequent than decreases upon cohesin depletion, which is opposite to what occurs at cohesin-binding active genes, where Pc decreases are 10-fold more frequent than increases (Figure 2C). Thus Rad21 depletion causes coincident PRC1 decreases at active cohesin-binding genes and PRC1 increases at silenced genes, where cohesin binding is generally restricted to Polycomb Response Elements (PREs; [12]). PRC1 binding may increase at silenced genes because it is released from active genes, increasing the amount available for binding. We cannot rule out the possibility, however, that cohesin directly competes with PRC1 for binding at genes marked by H3K27me3, but that under normal conditions, the cohesin levels fall below the threshold for detection by ChIP. It is unlikely that cohesin is epitope-masked at silenced genes, because it was not detected outside of PREs at silenced genes with antibodies against Smc1, SA, Rad21 and Nipped-B [12].

The antagonism between cohesin and PRC1 binding at PcG-silenced homeotic genes in the ANT-C and BX-C such as *Scr* and *Antp* (Figure 3) provides a molecular explanation for dominant suppression of *Pc* mutant homeotic phenotypes by cohesin mutations [10], [11]. The ectopic sex comb phenotype of heterozygous PRC1 mutants results from reduced silencing of *Scr*, and the abdominal segment transformations are caused by derepression of genes in the BX-C. We further confirmed the *in vivo* genetic antagonism by testing the effects of cohesin and releasin mutations on the phenotypes displayed by several PRC1 mutants. *Smc1* and *Rad21* cohesin mutations suppressed these phenotypes and *pds5* releasin mutations enhanced them, supporting the idea that cohesin antagonizes PRC1 function at silenced genes during development (Table S4).

The antagonism between cohesin and PRC1 binding at silenced genes predicts that heterozygous cohesin mutants would show phenotypic transformations opposite to those exhibited by PRC1 mutants because they would increase PcG silencing of homeotic genes. Indeed, we find that adult male flies heterozygous for *Rad21* (*vtd*) loss-of-function mutations exhibit mild and weakly penetrant posterior to anterior abdominal transformations resulting in lighter pigmentation of abdominal segment A5, which is an A5 to A4 transformation opposite to the A4 to A5 anterior to posterior transformation caused by PRC1 subunit mutations (Figure S9). The penetrance and extent of this transformation is increased by heterozygous *Nipped-B* kollerin mutations (Figure S9). This transformation indicates increased silencing of genes in BX-C, consistent with the increased PRC1 levels at these genes upon cohesin depletion in BG3 cells.

### PRC1 Influences RNA Polymerase Function at Both PcG-Silenced and Active Genes

Cohesin selectively binds active genes in which transcriptionally-engaged RNA polymerase II (Pol II) pauses several nucleotides downstream of the transcription start site [31], [32]. One mechanism by which cohesin controls gene transcription is influencing the transition of paused Pol II to elongation. Cohesin depletion decreases the levels of total and elongating phosphorylated Pol II in the bodies of most cohesin-binding genes in BG3 cells, indicating that it often facilitates transition of paused Pol II to elongation [32]. Cohesin also hinders transition of paused Pol II to elongation at genes that are strongly repressed by cohesin, which include those rare genes such as *inv* and *en* in BG3 cells that have an extensive cohesin - H3K27me3 overlap [31], [32].

Because cohesin facilitates PRC1 binding to active genes, we tested if PRC1 participates in the control of the transition of paused Pol II to elongation at active genes by mapping Pol II genome-wide before and after depletion of Ph in BG3 cells. We performed ChIP for Pol II subunit Rpb3 to measure total Pol II. Paused Pol II transitions to elongation after phosphorylation by P-TEFb [33], and thus we also conducted ChIP for the Pol II Rpb1 subunit phosphorylated on the serine 2 residues of the heptapeptide repeats in the C terminal domain (Ser2P Pol II) to detect elongating Pol II. We correlated the effects of Ph depletion on Pol II occupancy with changes in mRNA levels measured by microarrays (Table S3). As described below, the results show that PRC1 influences transition of paused Pol II to elongation, but the effects of PRC1 depletion differ from those of cohesin depletion, indicating that cohesin has roles in controlling Pol II activity at active genes beyond facilitating PRC1 binding. They also confirm that PRC1 inhibits transcription of PcG-silenced genes.

**Figure 4:**
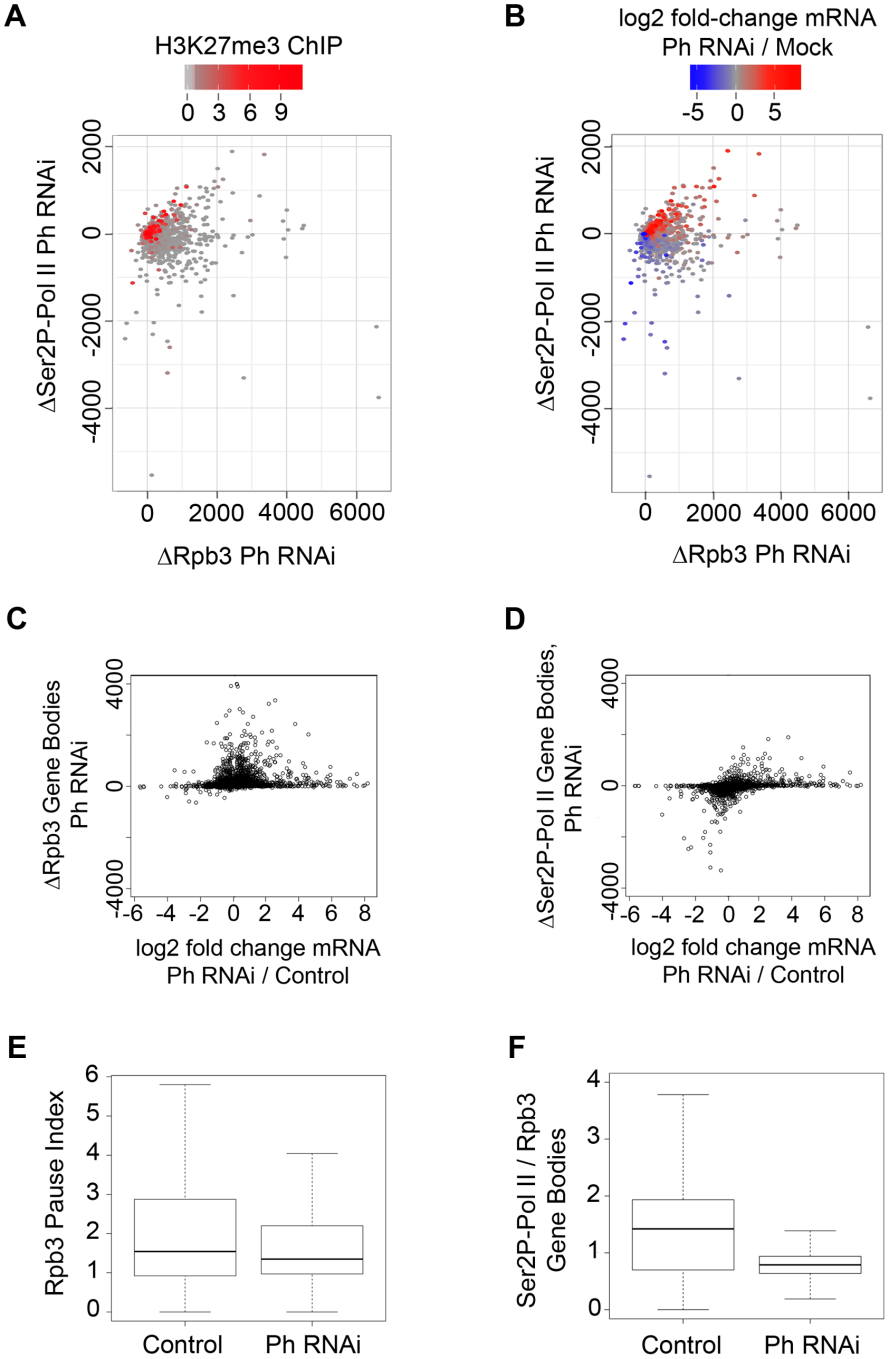
PRC1 affects Pol II occupancy and function at both silenced and active genes. (**A**) The change in total Pol II ChIP signal (ΔRpb3, Figure S7 method 1) in the bodies of the 13,132 genes measured for mRNA levels (X axis) plotted versus the change in elongating phosphorylated Pol II (ΔSer2P Pol II) (Y axis) caused by Ph (PRC1) depletion. Each gene is color-coded by a heat map to show the total H3K27me3 level in control cells. Many silenced genes show increases in both total and Ser2P Pol II, while the majority of active genes show increased total Pol II and reduced Ser2P Pol II. (**B**) Same plot as in A, except the genes are color-coded to show the change in mRNA level. Many silenced genes increase in expression, consistent with an increase in Ser2P Pol II, and many active genes decrease in expression, consistent with reduced Ser2P Pol II. (**C**) Change in total Pol II plotted versus the change in mRNA level after Ph depletion. Many genes show modest to large increases in total Pol II, but can show either reduced or increased mRNA levels. (**D**) Change in Ser2P Pol II ChIP signal plotted versus change in mRNA level (log2) for 13,132 genes after Ph depletion. Changes in Ser2P Pol II correlate with changes in mRNA levels. (**E**) Box plots showing the Pause Index (total Pol II density in the 400 bp region surrounding the transcription start site divided by the total Pol II density in the gene body, Figure S7 method 1) for all annotated genes before and after Ph depletion. (**F**) Box plots showing the ratio of Ser2P Pol II to total Pol II (Rpb3) in the bodies of all annotated genes before and after Ph depletion.

Plotting the change in Rpb3 levels versus the change in Ser2P Pol II levels shows that Ph depletion increased both Rpb3 and Ser2P Pol II at many PcG-silenced genes marked by H3K27me3, consistent with the idea that PRC1 is essential for PcG-silencing (Figure 3; Figure 4A; Figure S8). The increase in Ser2P Pol II is often accompanied by an increase in mRNA (Figure 4B,D).

By contrast, at a large fraction of active genes, which lack H3K27me3, Ph depletion increased total Pol II (Rpb3) in the gene body, but decreased Ser2P Pol II (Figure 3; Figure 4A; Figure S8). The genes with increased total Pol II and decreased Ser2P Pol II often show decreased mRNA (Figure 4B,C). Total Pol II increases and Ser2P Pol II decreases were much more frequent at cohesin-binding genes (40–50%) than at genes that lack cohesin (12–15%), and at Pc-binding genes (30–40%) than at genes that lack Pc (8–15%) (Figure S10).

Together, the findings that the total Pol II increases and Ser2P decreases upon Ph depletion (a) occur at a large fraction of active cohesin and Pc-binding genes, (b) happen more rarely at genes that don't bind cohesin, and (c) opposite changes (Ser2P Pol II increases or Rpb3 decreases) are rare, are compelling evidence that most of these Pol II changes are direct, and not caused indirectly by altered cell identity. Changes in cellular identity would be expected to affect a smaller fraction of active genes, and to alter Pol II occupancy more frequently in the opposite direction at cohesin and PRC1-binding genes and at non-binding genes.

In summary, although total Rpb3 increased at both silent and active genes upon PRC1 depletion and rarely decreased, Ser2P Pol II and mRNA often increased at PcG-silenced genes and frequently decreased at active genes (Figure 4A–D). The increase in total Pol II and decrease in Ser2P Pol II at many cohesin-binding active genes upon Ph PRC1 depletion argues that PRC1 blocks the release of non- or under-phosphorylated Pol II into elongation. Increases in total Pol II occurred 2.5-fold less frequently at promoters than in gene bodies upon Ph depletion, and the fold-increases in Pol II at promoters are also smaller (Figure S10). This argues that in most cases, transition to elongation, not Pol II recruitment or transcription initiation, is the key step regulated by PRC1 at active genes. Because promoter Pol II increases are more frequent than decreases, there may be modest effects on recruitment or initiation at some genes. The idea that PRC1 controls primarily the transition to elongation predicts that the pausing index, which is the ratio of total Pol II at the promoter to the total Pol II in the gene body will decrease upon Ph depletion. It also predicts that the ratio of phosphorylated Pol II to total Pol II in the gene body will decrease. Indeed, measuring the pause index and Ser2P to total Pol II ratio for all genes confirms global decreases in both cases, with a particularly dramatic decrease in the phosphorylated to total Pol II ratio in gene bodies (Figure 4E,F). The strong decrease in the fraction of phosphorylated Pol II is consistent with the observed decreases in mRNA, because Pol II phosphorylation is required for association of elongation and RNA processing factors with elongating Pol II [34]. The under-phosphorylated Pol II that enters the gene bodies upon PRC1 depletion may have reduced processivity, and the nascent RNA is also unlikely to be efficiently spliced or polyadenylated.

The effects of PRC1 depletion on Pol II activity at cohesin-binding genes differ substantially from those of cohesin depletion. Upon cohesion depletion, total Pol II and Ser2P Pol II levels usually change in the same direction, not in the opposite direction, with decreases being substantially more frequent than increases [32]. Thus the increase in total Pol II in the bodies of cohesin-binding genes upon PRC1 depletion likely requires cohesin, consistent with the finding that cohesin frequently facilitates the transition of paused Pol II to elongation [32]. Because the effects of cohesin depletion cannot be uncoupled from changes in transcription, we cannot rule out the possibility that the decreases in PRC1 upon cohesin depletion are caused in part by transcriptional changes instead of reduced recruitment or stabilization of PRC1 binding. We note, however, that the effects of cohesin reduction on PRC1 binding do not fully mirror the effects on Pol II occupancy, with the effects on PRC1 being more strongly skewed to the promoter. For instance, Rad21 depletion decreases Rpb3 at 12% of all active promoters [32] but decreases Pc on 18% of promoters, even though the starting Rpb3 signals are generally higher than Pc signals at promoters. In contrast, Rad21 depletion decreases Rpb3 in 50% of all active gene bodies, but Pc only in 35%. Thus reductions in Pol II levels at the promoter cannot explain all cases of reduced PRC1.

### Cohesin Depletion Decreases Long-Range Interactions between Polycomb Response Elements (PREs) at the *invected-engrailed* Gene Complex in BG3 Cells

**Figure 5:**
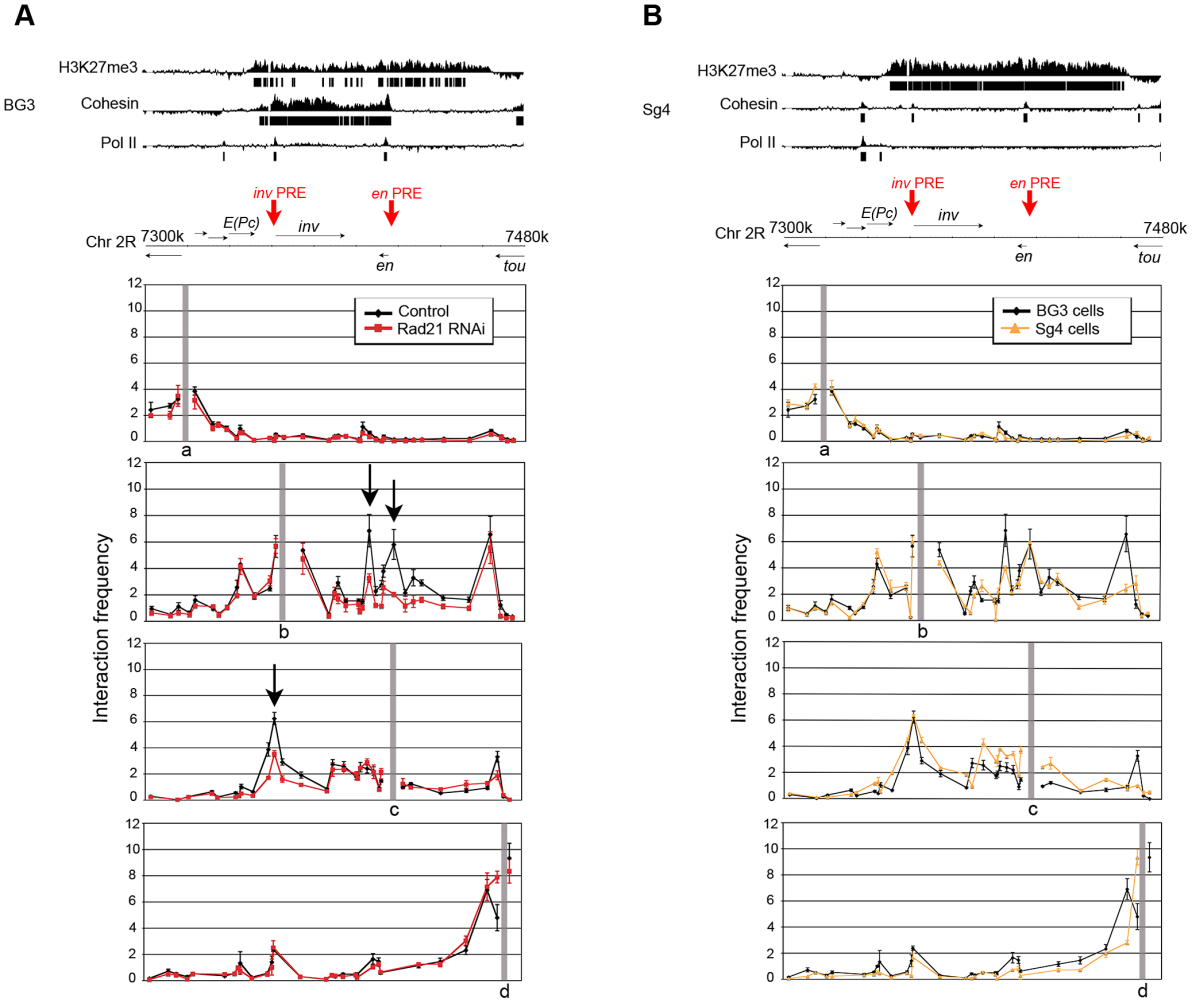
Cohesin depletion reduces long-range interactions between PRE-containing regions in the ***invected-engrailed*** **locus in BG3 cells.** Looping interactions were measured by chromosome conformation capture (3C). (**A**) The top is a diagram of the *inv-en* locus with ChIP-chip tracks for H3K27me3 and cohesin, which is aligned with the 3C panels below to show the anchor locations. Anchors **a** and **d**, shown in the top and bottom panels, are outside of the complex and show high local interactions but do not interact with points inside the complex. Anchor **b** (second panel) in the PRE-containing region upstream of *inv*, interacts with the regulatory region between *inv* and *en*, the *en* PRE/promoter region, and the end of the regulatory region near the 3′ end of the *tou* gene. Anchor **c** (third panel) near the *en* PRE, interacts with the PRE region upstream of *inv*, the regulatory region between *inv* and *en*, and with the end of the regulatory region near *tou*. In Rad21 depleted cells (red), mRNA levels increased 20 to 40-fold [13], consistent with several independent experiments [31], [32] and the looping between the *inv* PRE and the *en* PRE and regulatory region between the genes is decreased (arrows). Each interaction was measured with at least two biological replicates and two RT-PCR reactions per replicate. Error bars are the standard error of all RT-PCR replicates. (**B**) 3C analysis in Sg4 cells (yellow lines), in which *inv-en* is PcG-silenced, compared to the looping in BG3 cells (black lines).

In anterior wing disc (Figure 1) and cultured Sg4 cells [13], the *inv-en* complex is PcG-silenced and has high H3K27me3 and PRC1, but low cohesin. In the posterior wing disc, *inv-en* is expressed, with low H3K27me3, but high cohesin and PRC1 (Figure 1). In BG3 cells, the *inv-en* complex has a different cohesin-PcG structure than either the silenced or active states [13]. H3K27me3 marks the entire complex, including the large flanking regulatory region, but *inv* and *en* and the regulatory region between them also bind cohesin (Figure 5A). In these cells, *inv* and *en* mRNAs are present at modest levels and increase dramatically upon cohesin or PRC1 depletion [13], [31], [32]. Thus the *inv-en* complex in BG3 cells has a cohesin-PcG “restrained” state distinct from both the silenced and active states.

Cohesin depletion can reduce long-range looping between enhancers and promoters, and between CTCF-binding sites in mammalian cells [4], [35]. We thus considered the idea that part of the mechanism by which cohesin represses *inv-en* in BG3 cells is by facilitating looping between the Polycomb Response Elements (PREs) that recruit PcG complexes. There is a bipartite PRE just upstream of the *en* promoter, and another upstream of *inv* (Figure 5; [36]). Other regulatory sequences include enhancers upstream of each gene, between the genes, and throughout the long region extending from *en* to the *tou* gene [37].

We used chromosome conformation capture (3C) [38] to determine if the two PRE-containing regions in *inv-en* interact in BG3 cells, and if cohesin depletion reduces these interactions. We used four anchor restriction sites in a 180-kilobase region encompassing the *inv* and *en* genes, the regulatory regions, and flanking DNA (Figure 5). Two anchors (a, d) are outside the complex, and one (c) includes the *en* promoter and PREs. The fourth (b) is upstream of *inv* in the region containing a PRE.

Figure 5A shows that the *en* PRE-promoter anchor (c) interacts with the *inv* PRE region. Similarly, the anchor near the *inv* PRE (b) interacts with the *en* PRE and with the region between *inv* and *en*, which harbors tissue-specific enhancers [37]. Both PRE anchors (b, c) also interact with the ends of the *inv-en* complex demarcated by the ends of the H3K27me3 domain. In contrast, the external anchors (a, d) interact only with their immediately surrounding regions, and not with sites in the complex.

Upon depletion of the Rad21 cohesin subunit, which dramatically increased *inv* and *en* mRNA levels 20 to 40-fold [13] interaction between the *inv* and *en* PREs decreased substantially (Figure 5A). Interaction of the *inv* PRE-containing region with the enhancer-containing region between *inv* and *en* also decreased, although the enhancer-containing region has lower, but significant cohesin levels. It may be that this interaction is stimulated secondarily by PRE-PRE looping. Facilitation of PRE-PRE looping by cohesin may explain why cohesin represses *inv-en* in BG3 cells. We cannot formally exclude, however, the possibility that cohesin represses by a different mechanism, and that the increased transcription caused by cohesin depletion reduces the PRE-PRE interaction.

In Drosophila Sg4 cells in which *inv-en* is fully PcG-silenced, cohesin binds only at the two PREs, and there is no detectable Pol II or transcripts (Figure 5B; [12], [13]). We see interactions between the PREs as in BG3 cells, indicating that the PRE-PRE interactions seen in BG3 cells are consistent with PcG silencing (Figure 5B). Interaction of the *inv* PRE with the end of the regulatory domain is lower than in BG3 cells. We were unable to test if cohesin depletion reduced the PRE-PRE interactions in the fully silenced state because Sg4 cells are relatively refractory to RNAi treatment [13]. However, cohesin also occupies all known PREs in the silenced BX-C in BG3 cells [12], consistent with the possibility that cohesin can facilitate PRE-PRE looping at silenced genes.

## Discussion

**Figure 6:**
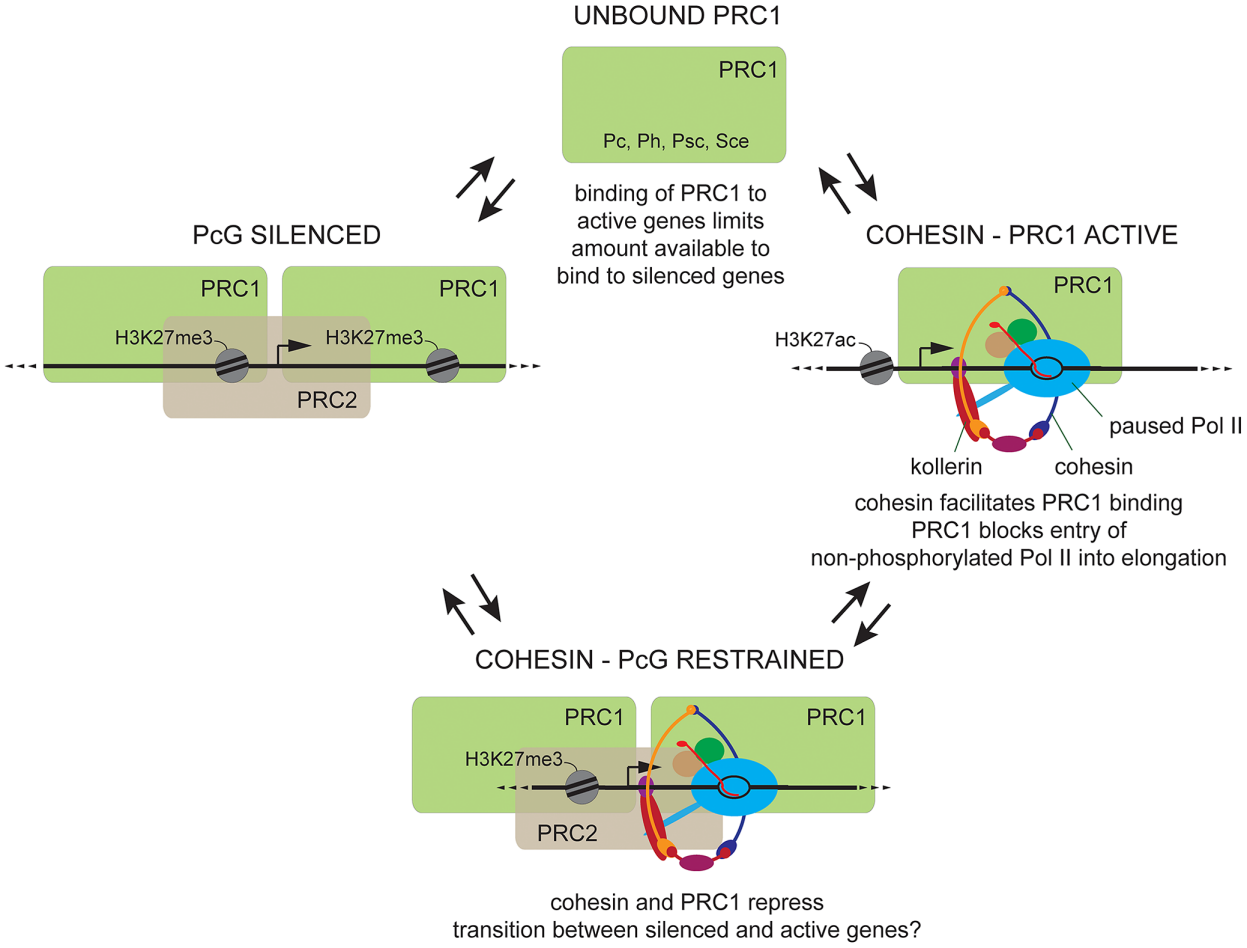
Three cohesin-PcG states and their interactions. In the PcG-silenced state (**left**), PRC1 (green rectangle) and PRC2 (tan rectangle) are both present, and the target genes are marked by the H3K27me3 histone modification made by PRC2. There is little detectable cohesin outside of Polycomb Response Elements (PREs). Depletion of cohesin increases PRC1 binding, and *vice versa*, and PRC1 depletion usually increases total Pol II, phosphorylated Pol II, and mRNA. The rare cohesin-PcG restrained state (**bottom center**) in which cohesin, PRC1 and PRC2 are all present may be a transition state between the silenced state and the cohesin-PRC1 active state (**right**). In the restrained state, cohesin and PRC1 both repress transcription, but the genes are not fully silenced. At the restrained *inv-en* gene complex in BG3 cells, cohesin depletion reduces long-range interactions between two Polycomb Response Elements (PREs), suggesting that cohesin represses by facilitating PRE-PRE looping. The cohesin-PRC1 active state (**right**) has promoter-proximal paused RNA Pol II with a short nascent RNA (red), PRC2 and H3K27me3 are absent, and cohesin aids PRC1 binding. Cohesin is the multi-subunit ring (orange, blue, purple) around the DNA. DSIF (tan circle) and NELF (green circle) are pausing factors, which along with the Pol II tail are phosphorylated by P-TEFb to induce transition to elongation. PRC1 depletion increases total Pol II but decreases elongating phosphorylated Pol II (Ser2P Pol II), with a corresponding decrease in mRNA. We thus posit that PRC1 prevents paused Pol II from entering into elongation until it is fully phosphorylated by P-TEFb. We theorize that binding of PRC1 to active genes limits the amount of unbound PRC1 (**top**) available to bind to PcG-silenced genes. This explains the *in vivo* genetic antagonism between cohesin and PRC1 in gene silencing, and how cohesin depletion simultaneously decreases PRC1 levels at active genes and increases them at silenced genes.

Our genome-wide studies in developing wing discs and cultured cells reveal three distinct cohesin-PcG states at their target genes in Drosophila, all of which occur at the *invected-engrailed* (*inv-en*) complex in different cell types. In Sg4 cells [12], [13] and anterior wing disc, *inv-en* is PcG-silenced and has high H3K27me3 showing PRC2 activity, high PRC1, and low or undetectable cohesin, except at the PREs (Figure 6, PcG silenced). This pattern also occurs at virtually all PcG-silenced genes in wing discs and BG3 cells. In this state, cohesin and PRC1 binding are mutually antagonistic in BG3 cells, consistent with the *in vivo* genetic antagonism between cohesin and PRC1 in determining segmental identity. Reducing cohesin increases silencing, while reducing PRC1 decreases silencing.

In BG3 cells, *inv-en* has the rare restrained state in which H3K27me3 (PRC2), PRC1 and cohesin overlap over long extended regions of several kilobases (Figure 6, cohesin – PcG restrained). The genes are not silenced, but depletion of either cohesin or PRC1 causes a large increase in transcription and mRNA [13], [31], [32]. In this case, cohesin inhibits transition of paused Pol II to elongation at a step distinct from those controlled by the DSIF and NELF pausing factors [31], [32]. As shown here, cohesin depletion reduces interactions between the two *inv-en* PREs in this state, suggesting that it represses by facilitating PRE-PRE looping. Although there are other genes with the cohesin-PcG restrained state in BG3 and Sg4 cells [13], there is currently no unambiguous evidence that this state occurs *in vivo*. It is possible that this rare state occurs at genes that are in transition from a silenced to active state or *vice versa*, but it may also be a stable state that serves to moderate gene expression.

Unexpectedly, we also found that PRC1 is present at the active *inv-en* complex in posterior wing disc, and at most other active cohesin-binding genes in both developing wing disc and BG3 cells (Figure 6, cohesin-PRC1 active). These genes lack H3K27me3, indicating that PRC2 is not present or active. Cohesin facilitates PRC1 binding to active genes in BG3 cells, and PRC1 depletion decreases the ratio of phosphorylated to total Pol II in the gene body and reduces mRNA levels. This finding greatly expands the known gene regulatory functions of both cohesin and PRC1 and provides new insights into the mechanisms by which they control transcription.

As outlined in Figure 6, we propose that the three cohesin-PcG states are functionally linked. We posit that cohesin facilitates binding of PRC1 to active genes via direct interactions, and that PRC1 then inhibits the transition of non-phosphorylated paused Pol II to elongation. We favor the idea that binding of PRC1 to active genes sequesters enough PRC1 at active genes to limit the amount of PRC1 available for binding to PcG-silenced genes targeted by PRC2. Although there are other possibilities, this explains why cohesin depletion simultaneously reduces PRC1 binding to active genes and increases binding to silenced genes. The cohesin-mediated sequestration of PRC1 at active genes can also explain the genetic antagonism between cohesin and PRC1 in silencing of the *Scr* gene and the BX-C *in vivo*. These and alternative ideas are discussed in more detail below.

### Cohesin – PRC1 Interactions at PcG-Silenced Genes

PcG-silenced genes are targeted by both PRC1 and PRC2, and generally do not bind measurable levels of cohesin except at PREs, but cohesin reduction increases Pc levels at PcG-silenced genes and PRC1 depletion increases cohesin binding. This antagonistic relationship contrasts with the functional interactions between PcG proteins and the Trithorax (Trx) protein, which binds PREs of silenced genes and is generally antagonistic to PcG silencing, but whose knockdown does not alter PRC1 binding [25].

Multiple mechanisms, which are not mutually exclusive, could contribute to the antagonism between PRC1 and cohesin association with PcG-silenced genes. Upon Ph PRC1 subunit depletion, increased transcription could facilitate cohesin binding. It might be expected, based on their direct interaction, that PRC1 would recruit cohesin to silenced genes, but PRC1 also has negative effects on cohesin binding at many active genes, and the presence of PRC2 at silenced genes may enhance these effects. For example, binding of Pc to H3K27me3 at silenced genes could alter PRC1 conformation such that the PRC1-cohesin contacts inhibit cohesin loading by kollerin, and/or facilitate cohesin removal by releasin.

Cohesin depletion increases PRC1 levels at PcG-silenced genes, even though cohesin binding is very low at these genes outside of the PREs. One idea is that a reduction in cohesin dosage could release PRC1 from active genes, thereby making more PRC1 available to bind to silenced genes (Figure 6). Alternatively, cohesin and PRC1 binding could be dynamically competitive at silenced genes, with the competition favoring PRC1, making it difficult to detect cohesin. Both models are consistent with the finding that a dominant *wapl* releasin mutation, which globally increases and stabilizes cohesin binding, causes similar mutant phenotypes as PRC1 mutations, indicating reduced PRC1 at *Scr* and other silenced genes [9]. Higher cohesin levels at active genes in the releasin mutant could sequester more PRC1, making less available for binding to silenced genes. It more difficult to explain how more cohesin binding at silenced genes could directly reduce PRC1 binding in this mutant. One possibility is that binding of the Pc PRC1 subunit to H3K27me3 at silenced genes alters PRC1 conformation in a manner that allows cohesin to stimulate PRC1 removal instead of facilitate its binding.

### Role of PRC1 in Transcription of Active Cohesin-Bound Genes

Because cohesin directly interacts with PRC1 and cohesin depletion reduces Pc binding at active genes, we posit that cohesin directly recruits and/or stabilizes PRC1 binding to active genes. As discussed below, our studies reveal that PRC1 controls Pol II phosphorylation and transition of paused Pol II to elongation at active genes. This may be similar to the function of PRC1 at some silenced and bivalent genes. Although the mechanisms by which PcG complexes repress transcription are not well understood, in some contexts, they inhibit transition of paused transcriptionally-engaged Pol II to active elongation [39]–[41].

Our findings suggest that at active genes, PRC1 facilitates phosphorylation of Pol II to the Ser2P Pol II elongating form and/or blocks entry of non-phosphorylated paused Pol II into elongation. Cohesin preferentially binds genes with promoter-proximal paused Pol II, and often facilitates, or less frequently, inhibits transition of paused Pol II to elongation [31], [32]. Transition of paused Pol II to elongation requires phosphorylation of the NELF and DSIF pausing factors and Pol II by P-TEFb [33]. At most cohesin-binding genes, cohesin depletion reduces the levels of both total and phosphorylated Pol II in the gene body [32]. Here we find that PRC1 depletion, similar to cohesin depletion, reduces Ser2P Pol II at many active genes, but opposite to cohesin depletion, increases total Pol II in the same gene bodies. Thus both the global pausing index and ratio of phosphorylated Pol II to total Pol II in gene bodies decrease upon PRC1 depletion. This argues that Pol II enters into elongation with inadequate phosphorylation. One possible mechanism could be that PRC1 helps NELF and DSIF pausing factors restrain Pol II at the promoter until it is fully phosphorylated.

The decrease in the ratio of phosphorylated to total Pol II upon PRC1 depletion is accompanied by a decrease in mRNA at many genes. This argues that the under-phosphorylated Pol II is less processive, and/or that the nascent RNA is not efficiently processed to mRNA. This is expected, because Pol II phosphorylation, in addition to facilitating transition to elongation, is required for binding of RNA processing factors to the transcription complex [34].

### Other Evidence for PcG Function at Active Genes

The fact that PRC1 depletion affects Pol II activity and mRNA levels at a large fraction of active cohesin-binding genes confirms that PRC1 is present and plays an important role at these genes. The finding that PRC1 directly controls transcription of many active genes is surprising because it is generally thought that PRC1 functions primarily at PcG-silenced genes. There is, however, published evidence consistent with our findings. Müller and colleagues used chromatin from mixed imaginal discs to perform Ph ChIP-chip with a different antibody than the one used here [28], [42]. Although the mixture of many different cell types creates false overlaps, close examination of their data reveals that Ph associates with many constitutively active genes, including *Act5C* and some ribosomal protein genes, as we found in both wing discs and BG3 cells. Moreover, using different Pc, Psc and Ph antibodies, Paro and colleagues found that PRC1 preferentially binds promoters with paused RNA polymerase in cultured S2 cells [43]. Although the role of PRC1 in paused Pol II function was not investigated, this is consistent with the selective association of cohesin with genes that have paused Pol II [31], [32] and our finding here that cohesin aids binding of PRC1 to active genes.

There is also evidence that PcG complexes regulate some active genes in mammalian cells. In differentiated T helper cells, both PRC1 and PRC2 components are required for transcriptional activation of cytokine genes, although they repress HOX genes in the same cells [44]. Some expressed genes exhibit H3K27me3, and the Ezh1 alternative methyltransferase is in PRC2 complexes that promote transcription during myogenic differentiation [45], [46]. Ezh1 knockdown decreases Ser2P Pol II at the genes that it binds, similar to our findings with Ph depletion. We did not find any evidence, however, that PRC2 promotes transcription of active genes in Drosophila, which unlike mammals, has only one E(z) methyltransferase.

The work presented here clearly establishes functional connections between cohesin and PRC1, and suggests mechanisms for how cohesin and PRC1 control each other's activities and gene transcription. Our finding that three different cohesin-PcG states can exist at the *invected-engrailed* gene complex in different cells raises the question of how these various states arise. The presence or absence of PRC2 is likely to be one important factor that determines whether cohesin and PRC1 compete or collaborate, but the factors responsible for establishing these distinct states and controlling transitions between them remain to be determined.

## Materials and Methods

### Drosophila Culture and Genetic Crosses

Drosophila was cultured and genetic crosses were conducted at 25° as previously described [17]. The *w^1118^; P{en2.4-GAL4}e16E, P{UAS-myr-mRFP}1, P{NRE-EGFP.S}5A* line [21] was used for preparing chromatin from anterior and posterior wing imaginal discs. Stocks were obtained from the Bloomington stock center.

*Nipped-B*, *pds5*, and cohesin mutant alleles have been described previously [10], [17], [47]. PcG mutant stocks were obtained from the Bloomington stock center and Rick Jones (SMU). At least 30 male progeny from crosses between cohesin and PcG mutants were scored. Sex comb bristles on first, second, and third legs were counted, and abdominal transformations were scored using a defined arbitrary scale.

### ChIP-chip

Chromatin was prepared from late third instar Oregon-R wing imaginal discs according to Papp and Müller [48], without dialysis. Sixty to seventy-five discs were used per immunoprecipitation, or 100 to 120 anterior or posterior segments. Chromatin preparation from BG3 cells, immunoprecipitation, and Affymetrix Drosophila 2.0R genome tiling microarray hybridization were as previously described [12]. Rick Jones (SMU) generously provided Pc antibodies (Pc-RJ), and Vincenzo Pirrotta (Rutgers) kindly provided Pc (Pc-VP), Ph, and Psc antibodies. Renato Paro (ETH Zürich), Jürg Müller (Max Planck Institute of Biochemistry), and Giacomo Cavalli (Institute of Human Genetics) provided additional Pc and Ph antibodies. Karen Adelman (NIEHS) generously provided Rpb3 antibodies. Ser2P Pol II and H3K27me3 antibodies were purchased from Abcam (ab5095, ab6002) and 8WG16 Pol II antibody was purchased from Covance (MMS-126R).

MAT software [19] was used to calculate ChIP enrichment. MAT scores measure enrichment relative to an input control and scale linearly with the log2 IP/control ratio. MAT has been experimentally demonstrated to be more sensitive and quantitative than other algorithms for measuring ChIP enrichment with Affymetrix tiling arrays, providing sensitivity equivalent to ChIP-seq at a density of 1 aligned read per genome base pair [18], [49]. MAT uses probe sequence to perform within-array normalization, avoiding assumptions associated with quantile normalization.

### Genomic Data Analysis

Bed files showing significant enrichment of proteins mapped by ChIP-chip (Rad21, Nipped-B, H3K27me3, Pc-RJ, Pc-VP, Ph, Psc, Rpb3, Ser2P Pol II) at statistical thresholds of p≤10^−3^ or p≤10^−2^ were generated using MAT. Genes bound by a given protein were determined using the bed files and FlyBase gene annotations (www.flybase.org, v5.28) with the Intersect tool of Galaxy/Cistrome [50] or BEDTools [51]. Venn diagrams were generated using eulerAPE (www.eulerdiagrams.org/eulerAPE/). Gene-based ChIP-chip signal data was extracted from MAT score files and aligned to gene expression data using custom programs. Data was analyzed using Microsoft Excel and R ([52]; http://www.R-project.org). For some analyses, differences in integrated ChIP signals (MAT scores) over each annotated gene were calculated (Figure S6, method 1). For others, increases and decreases in Rad21, Pc, Rpb3 and Ser2P binding were measured by the difference in the ChIP MAT scores at each measured point in the genome between the RNAi treated samples and the controls (Figure S6, method 2). Increases or decreases ≥2 standard deviations from the genome-wide median difference that extend for at least three contiguous microarray features (105 bp) were used to generate bed files that were matched with gene annotations using BEDTools [51]. Examples of the increase (+) and decrease (−) bed files are shown in Figure 3. Based on changes in Pol II occupancy upon cohesin depletion, these methods agree closely with results obtained by ChIP-qPCR at selected genes and genome-wide PRO-seq analysis [31], [32].

### mRNA Quantification

Total RNA from wing discs or BG3 cells was isolated using Zymo ZR RNAi MicroPrep columns (Zymo Research). Genome-wide analysis of wild-type wing disc mRNA with four biological replicates was performed using Affymetrix Drosophila GeneChip 2.0 microarrays as previously described [13]. Genome-wide measurements of three biological replicates of control BG3 cells and BG3 cells treated with Ph RNAi (see below) for five days was conducted in the same manner.

### BG3 Cell Culture and RNAi

BG3 cells were cultured and proteins were RNAi-depleted for Rad21, Nipped-B or Ph as described [13]. The double-stranded RNAs used for Rad21 and Nipped-B RNAi were as described [13]. Two double-stranded RNAs targeting Polyhomeotic were used in concert; the constructs target regions of homology between *polyhomeotic proximal* and *polyhomeotic distal*. Primer sequences are as follows: Ph RNAi A, forward: TAATACGACTCACTATAGGGAGAAGCCATCAGCACCATGTCGC, reverse: TAATACGACTCACTATAGGGAGACGTAATTTCCGCCAGCGAATC. Ph RNAi B, forward: TAATACGACTCACTATAGGGAGATGCCCATTGATTCGCCCAAG, reverse: TAATACGACTCACTATAGGGAGATGCAACTTGTGGTAAAGGTGCC. These targets were designed using tools in the Drosophila RNAi Screening Center (DRSC) website (www.flyrnai.org/) to avoid off-target effects. All RNAi-treated chromatin and 3C samples were collected 5 days after RNAi treatment.

### Chromosome Conformation Capture (3C)

3C was conducted using a modification of the strategy outlined by Miele et al. [53]. 10^8^ BG3 or Sg4 cells were collected, washed with phosphate buffered saline, pH 7.6 (PBS) and then with hypotonic buffer [10 mM HEPES pH 7.9, 50 mM NaCl, 1 mM dithiothreitol (DTT), 10 mM MgCl_2_, protease inhibitor cocktail (cOmplete Mini, EDTA-Free Protease inhibitor tablets, Roche)]. Cells were lysed in hypotonic buffer containing 0.35 M sucrose and 0.2% NP-40, vortexed for one minute, and immediately cross-linked with 1% formaldehyde for 10 minutes at room temperature. Cross-linking was stopped by adjustment to 135 mM glycine, and nuclei were isolated by centrifugation onto a 0.8 M sucrose cushion. Nuclei were washed in EcoRI restriction enzyme buffer, and 0.1% SDS was added to extract free protein. 1% Triton X-100 was used to sequester SDS, and EcoRI digestions (500–700 units) were performed overnight at 37°. SDS was added to sequester EcoRI, and the digested DNA was ligated at 18° for 2 hours at a concentration of approximately 4 ng per microliter. Cross-linking was reversed by Proteinase K incubation at 65° for 6 hr or overnight, and DNA was isolated by sequential phenol, phenol-chloroform, and chloroform extractions, followed by precipitation with 0.5 volumes of 7.5 M ammonium acetate and 2.5 volumes of ethanol. DNA was dissolved in TE, digested with 500 ng per microliter RNAse A for 30 min at 37° and used for RT-PCR quantification.

Digestion and religation of BAC DNA containing the entire *inv-en* locus was used as a normalization control (BACR13F13; BACPAC Resources, Oakland, CA). This template was used to determine the amplification efficiency of each primer pair. EcoRI digestion efficiency was confirmed to be over 85% in several experiments using PCR primer pairs spanning the EcoRI sites. When the experiment was conducted without formaldehyde cross-linking, we were unable to detect 3C ligation products except between two immediate adjacent EcoRI sites (likely resulting from incomplete digestion).

### Accession Numbers

Genome-wide ChIP and expression data generated for this study is deposited in the GEO database (accession no. GSE42106).

## Supporting Information

Figure S1 Expression of invected and engrailed in dissected anterior and posterior 3^rd^ instar wing discs. The levels of *inv* and *en* RNAs were determined by RT-PCR relative to the *RpL32* transcripts using previously described primers [13]. Two independent dissections are shown. Error bars are standard errors of all RT-PCR replicates.
(TIF)

Figure S2 Validation of Pc-RJ and Ph antibodies and co-immunoprecipitation of PRC1 with cohesin. The left panel shows a western blot of whole cell extract of mock RNAi-treated BG3 cells and BG3 cells treated with Pc RNAi [13] for three days. The blot was probed with affinity-purified rabbit polyclonal anti-Pc [23] diluted 1∶2000 and anti-actin. The central panels show a western blot of whole cell extract of mock RNAi-treated BG3 cells and BG3 cells treated with Ph RNAi for five days. The blot was probed with affinity-purified rabbit polyclonal anti-Ph [22] diluted 1∶2000. The blot was stripped and reprobed with anti-actin antibody as a loading control (lower panel). The cross-reacting band marked with the asterisk (*) is not detected in nuclear extract. The right panel consists of western blots that show co-immunoprecipitation of Pc and Ph with cohesin. DNase I-treated nuclear extract from cultured Kc cells (4 mg protein per mL) previously used to show co-immunoprecipitation of cohesin subunits, was immunoprecipitated with guinea pig anti-Rad21 serum or preimmune serum as previous described ([54]; 10 micrograms of serum per 100 microliters of extract). The extract control lanes contained 0.6 microliter of the nuclear extract. The top set of lanes are all from the same blot probed with rabbit anti-Pc-RJ antibody at a dilution of 1∶2000, and the bottom set of lanes are probed with anti-Ph antibody diluted 1∶1200. Co-precipitation of the Smc1 cohesin subunit was confirmed by stripping and probing the blots with anti-Smc1 (not shown).
(TIF)

Figure S3 Correlations of PRC1 subunit enrichment in 3^rd^ instar wing discs. The panels show the plots of ChIP enrichment (MAT scores) for each microarray feature over a 400 kb region (chromosome 2L nt 2124748–2527439; 10,000 points) that includes the PcG-silenced *dpp* gene and several active genes for pairwise combinations of the different PRC1 antibodies used in this study. The numbers in red in the upper left corner of each panel is the genome-wide correlation coefficient for each pairwise comparison. Plots in which Pc-VP and Psc ChIP enrichment are plotted against Pc-RJ or Ph enrichment separate silenced and active genes into two distinct populations as indicated.
(TIF)

Figure S4 Binding of multiple Psc, Ph, Pc, Rad21, and Pol II to the active *dm/myc* gene in 3^rd^ instar wing discs. The genomic ChIP-chip tracks are as described in Figure 1. Bars underneath indicate binding called at p≤10^−3^.
(TIF)

Figure S5 ChIP-qPCR for Pc and Ph on cohesin-binding active genes. The bar graph shows the enrichment obtained by Pc and Ph ChIP-qPCR of wing disc chromatin at three active genes (*dm/myc*, *Rbf*, *RpL32*) that lack H3K27me3, a PcG-silenced gene (*Abd-B*) with H3K27me3, and three empty regions that lack genes, RNA Pol II, cohesin, PRC1, and H3K27me3 (2L_empty_A, 2L_empty_B, 2R_empty_B) relative to a fourth empty site (2R_empty_A). Error bars show the standard error for all PCR replicates. The primer sequences and genomic locations of the amplicons are given below the graph. The antibodies used [27], [28] are different from those used for genome-wide mapping.
(TIF)

Figure S6 Cell cycle analysis of BG3 cells depleted for Rad21 and Pc. The panels show fluorescence-activated cell sorting analysis of mock-RNAi treated BG3 cells, and BG3 cells depleted by ∼80% for Rad21 or Pc. The estimated percentages of cells in G1, G2 and S phase are indicated.
(TIF)

Figure S7 Methods used to measure changes in protein occupancy using ChIP-chip data. Both methods used the MAT scores calculated using at least two independent biological replicates for each measurement. This ChIP-chip method provides a highly quantitative and reproducible measure of binding. For instance, as shown in Figure S3, the genome-wide correlation between the Pc MAT scores in the anterior and posterior halves of the wing was 0.98, which compares two independent anterior chromatin preparations and ChIP experiments to two independent posterior chromatin preparations and ChIP experiments. In method 1, the MAT score for all microarray features contained within each annotated transcription unit, gene body, or promoter region were summed for the RNAi-depleted and mock control cells, and the total in the control for each gene was subtracted from the total in the RNAi-depleted cells to determine the total change in binding for each gene. To calculate the pause index, the median of the MAT scores in the promoter region was divided by the median in the gene body. In method 2, the MAT score for each microarray feature in the mock control cells was subtracted from the MAT score for each feature in the RNAi-depleted cells to generate a genome-wide array of Δ values for all microarray features. The distribution of these genome-wide Δ values were visualized by a histogram as shown on the right, and the mean and median values were calculated to ensure that both were close to zero, and that the distribution was close to normal. The standard deviation of this distribution was calculated (typically between 0.6 to 1 MAT unit), and then all regions in the genome in which the ΔMAT score deviated from the median Δ by at least two standard deviations for at least three microarray features in a row (typically 105 bp) were mapped to detect significant changes in binding. These regions were aligned with the genome annotation to determine which genes have a change in binding. In practice, most intervals were longer than 105 bp, and several occurred within the same gene, demonstrating high statistical significance. Rare cases of a gene scoring for both an increase and a decrease were resolved by visual inspection in a genome browser. Based on measuring changes in Pol II occupancy upon Rad21 depletion, these two methods agree well with ChIP-qPCR at selected genes [31], and genome-wide changes measured by precision global run-on sequencing (PRO-seq; [32]).
(TIF)

Figure S8 Examples of changes in Rad21 and Pol II levels upon Ph depletion, and changes in Pc binding upon Rad21 depletion at active and PcG-silenced genes. The left and middle panels show the active *Dsp1* transcription factor and *Lk6* protein kinase genes, and the right panel shows the PcG-silenced *bifid* (*bi*) transcription factor gene.
(TIF)

Figure S9 Abdominal transformations of cohesin mutants. Heterozygous *Rad21^36^* (*vtd^36^*) exhibits mild, partially penetrant (10 to 20%) A5 to A4 transformations. Arrows indicate regions with lighter than normal pigmentation. The representative left-most and central panels on the top show *Rad21^36^*/+ pigmentation phenotype close to wild-type, while the right-most is an instance where pigmentation is significantly reduced (arrow), indicating A5 towards A4 transformation. The transformation is significantly stronger in *Nipped-B^407^/+; Rad21^36^/+* trans-heterozygotes (bottom row) and penetrance is 100%.
(TIF)

Figure S10 Ph depletion preferentially alters Pol II occupancy at cohesin-PRC1 binding genes in BG3 cells. The top bar graph shows the percentage of genes that bind cohesin (red) or that don't bind cohesin (blue) that show an increase (UP) or decrease (DOWN) in Pol II occupancy upon Ph depletion. Cohesin binding was determined at p≤10^−3^, and changes in binding were determined by method 2 in Figure S7. The promoter was defined as the 200 bp region surrounding the annotated transcription start site, and the gene body was defined as the rest of the annotated transcription unit. The bottom panel shows the same scoring for genes that bind Pc (orange) or don't bind Pc (green). Pc binding was determined at p≤10^−3^. The box plot in the upper right shows that the fold-changes in total Pol II (Rpb3) density in the gene body (method 1, Figure S7) are usually larger than at promoters.
(TIF)

Table S1 Genome-wide correlation coefficients for ChIP signals in wing imaginal discs.
(DOC)

Table S2 Genome-wide data used in this study.
(DOC)

Table S3 Binding of cohesin and PRC1 and mRNA levels for annotated genes in wing discs and ML-DmBG3 cells. “1” indicates binding within the transcription unit called at p≤10^−3^ and 0 indicates no binding. Expression values are in log2 units.
(XLS)

Table S4 Genetic interactions between heterozygous sister chromatin cohesion and PRC1 subunit mutations.
(DOC)
