# Supplementary figures and images for: Cohesin and Polycomb Proteins Functionally Interact to Control Transcription at Silenced and Active Genes
Source: PLoS Genet. 2013 Jun 20;9(6):e1003560. doi: 10.1371/journal.pgen.1003560 (PMC3688520; doi:10.1371/journal.pgen.1003560)

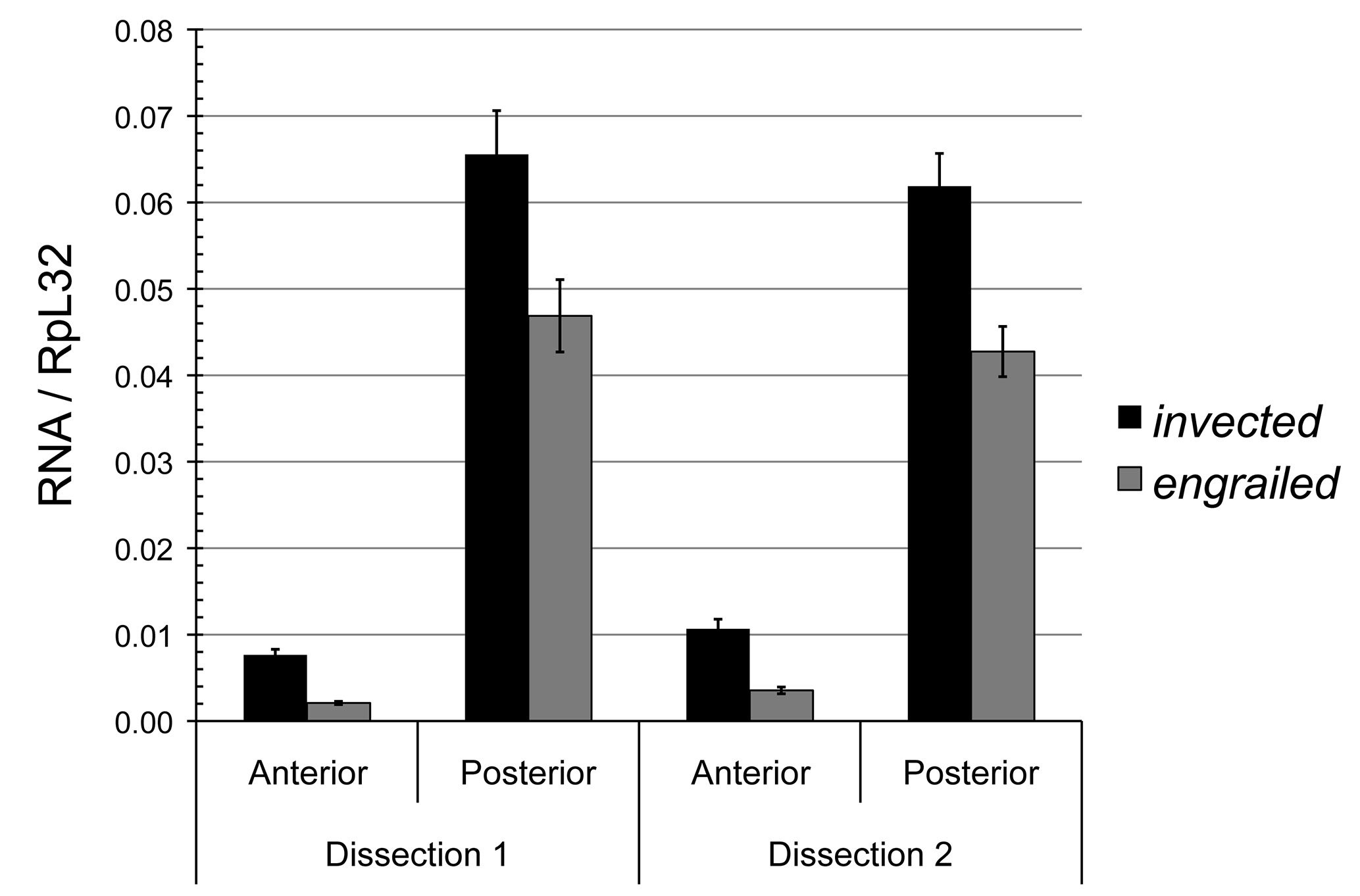

Supplement: Figure S1 — Expression of invected and engrailed in dissected anterior and posterior 3rd instar wing discs. The levels of inv and en RNAs were determined by RT-PCR relative to the RpL32 transcripts using previously described primers [13]. Two independent dissections are shown. Error bars are standard errors of all RT-PCR replicates. (TIF) [file pgen.1003560.s001.tif]

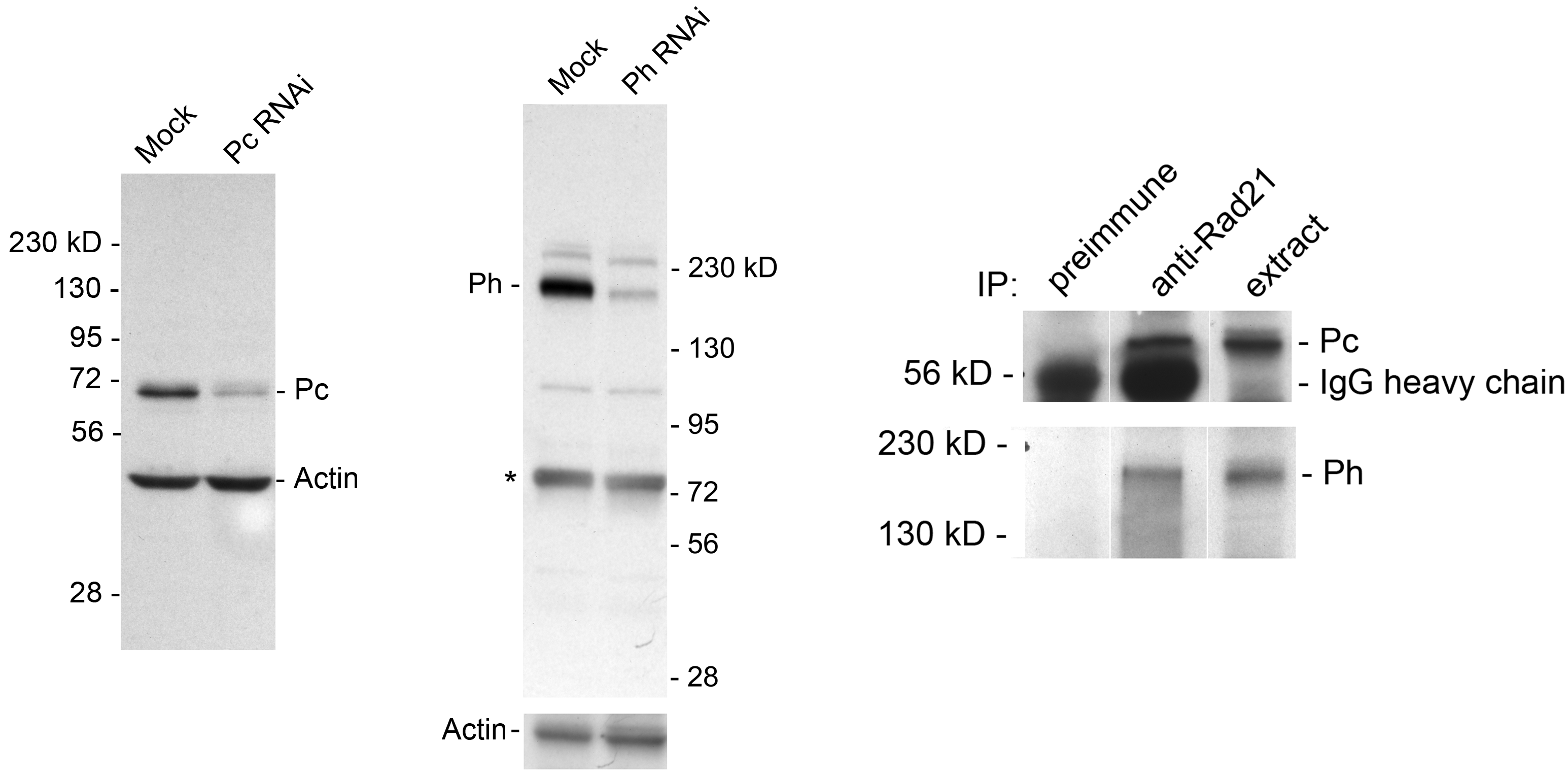

Supplement: Figure S2 — Validation of Pc-RJ and Ph antibodies and co-immunoprecipitation of PRC1 with cohesin. The left panel shows a western blot of whole cell extract of mock RNAi-treated BG3 cells and BG3 cells treated with Pc RNAi [13] for three days. The blot was probed with affinity-purified rabbit polyclonal anti-Pc [23] diluted 1∶2000 and anti-actin. The central panels show a western blot of whole cell extract of mock RNAi-treated BG3 cells and BG3 cells treated with Ph RNAi for five days. The blot was probed with affinity-purified rabbit polyclonal anti-Ph [22] diluted 1∶2000. The blot was stripped and reprobed with anti-actin antibody as a loading control (lower panel). The cross-reacting band marked with the asterisk (*) is not detected in nuclear extract. The right panel consists of western blots that show co-immunoprecipitation of Pc and Ph with cohesin. DNase I-treated nuclear extract from cultured Kc cells (4 mg protein per mL) previously used to show co-immunoprecipitation of cohesin subunits, was immunoprecipitated with guinea pig anti-Rad21 serum or preimmune serum as previous described ([54]; 10 micrograms of serum per 100 microliters of extract). The extract control lanes contained 0.6 microliter of the nuclear extract. The top set of lanes are all from the same blot probed with rabbit anti-Pc-RJ antibody at a dilution of 1∶2000, and the bottom set of lanes are probed with anti-Ph antibody diluted 1∶1200. Co-precipitation of the Smc1 cohesin subunit was confirmed by stripping and probing the blots with anti-Smc1 (not shown). (TIF) [file pgen.1003560.s002.tif]

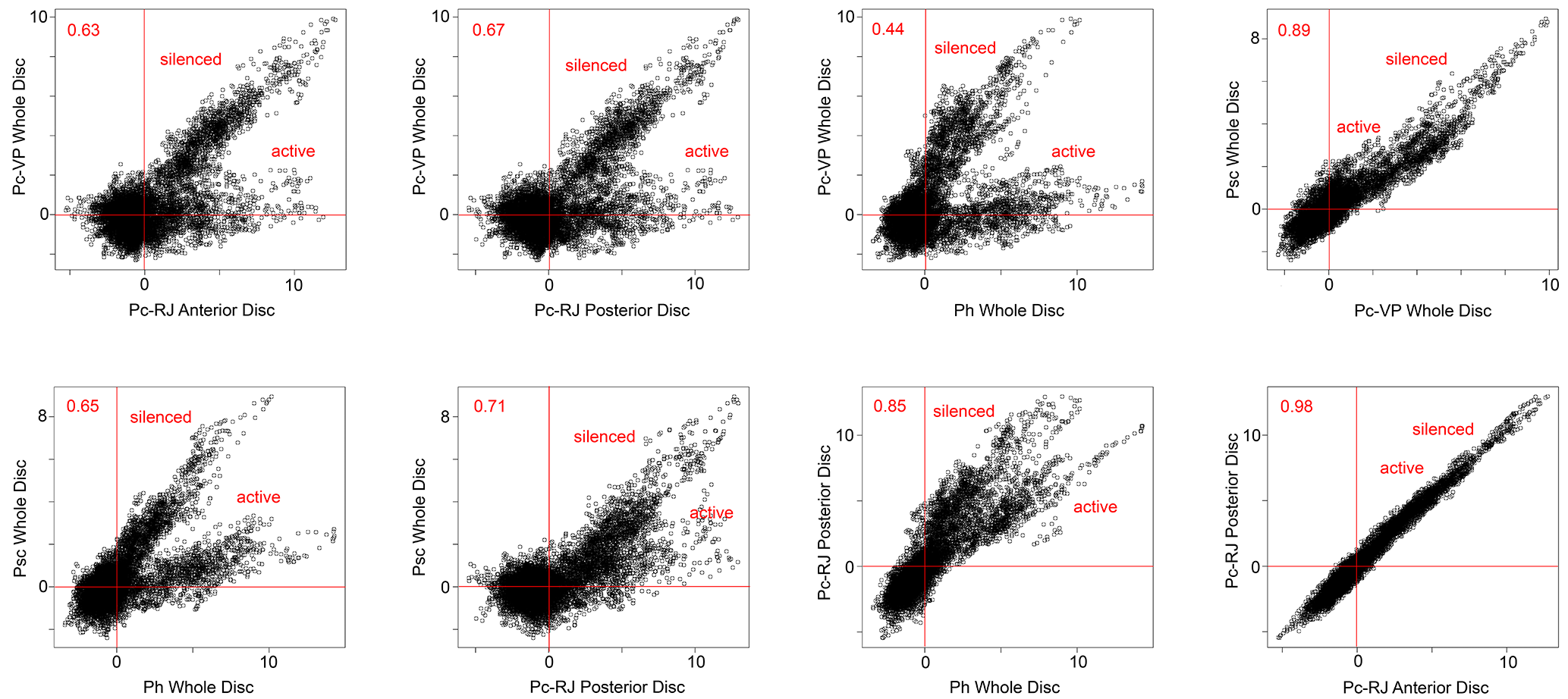

Supplement: Figure S3 — Correlations of PRC1 subunit enrichment in 3rd instar wing discs. The panels show the plots of ChIP enrichment (MAT scores) for each microarray feature over a 400 kb region (chromosome 2L nt 2124748–2527439; 10,000 points) that includes the PcG-silenced dpp gene and several active genes for pairwise combinations of the different PRC1 antibodies used in this study. The numbers in red in the upper left corner of each panel is the genome-wide correlation coefficient for each pairwise comparison. Plots in which Pc-VP and Psc ChIP enrichment are plotted against Pc-RJ or Ph enrichment separate silenced and active genes into two distinct populations as indicated. (TIF) [file pgen.1003560.s003.tif]

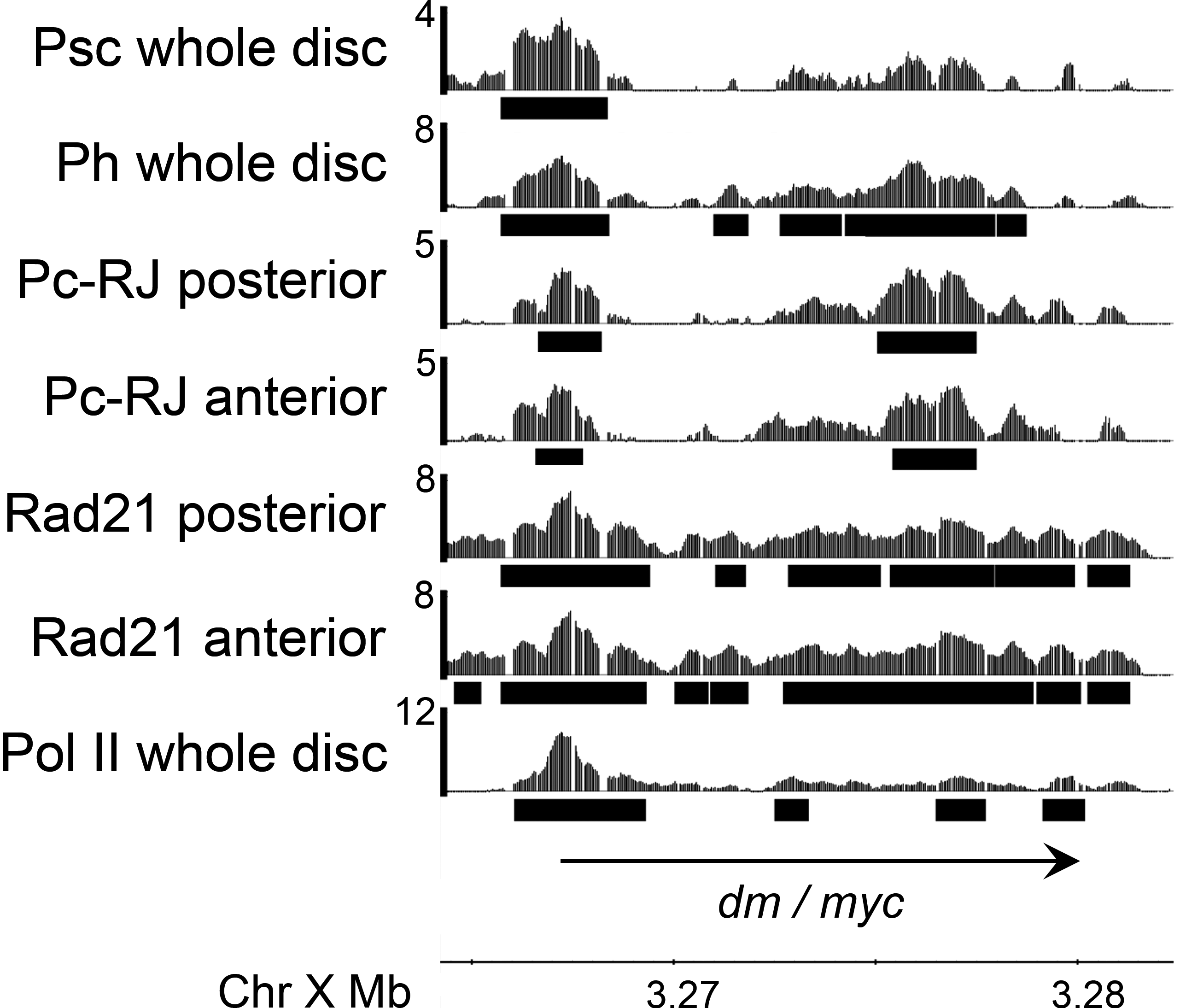

Supplement: Figure S4 — Binding of multiple Psc, Ph, Pc, Rad21, and Pol II to the active dm/myc gene in 3rd instar wing discs. The genomic ChIP-chip tracks are as described in Figure 1. Bars underneath indicate binding called at p≤10−3. (TIF) [file pgen.1003560.s004.tif]

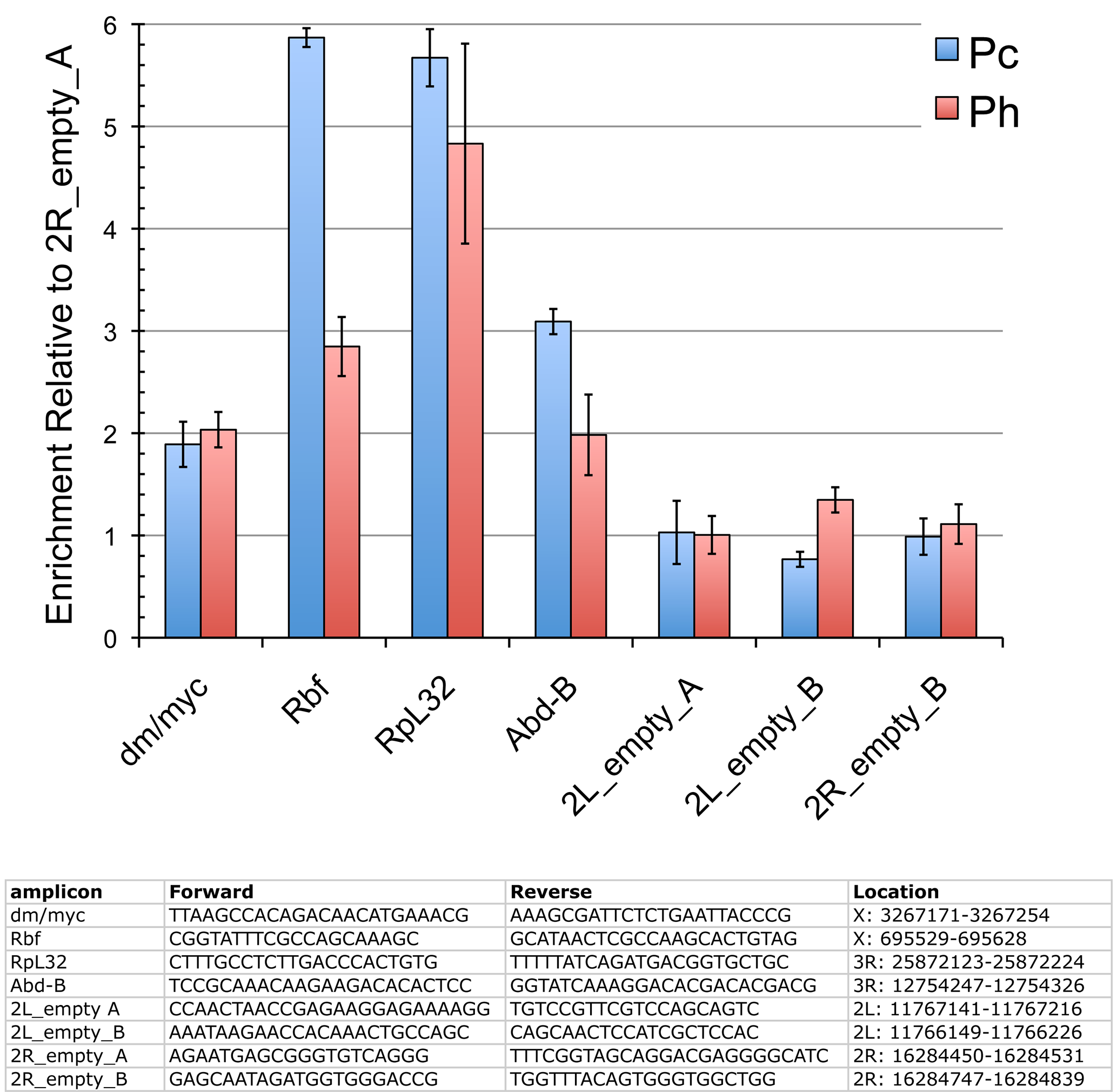

Supplement: Figure S5 — ChIP-qPCR for Pc and Ph on cohesin-binding active genes. The bar graph shows the enrichment obtained by Pc and Ph ChIP-qPCR of wing disc chromatin at three active genes (dm/myc, Rbf, RpL32) that lack H3K27me3, a PcG-silenced gene (Abd-B) with H3K27me3, and three empty regions that lack genes, RNA Pol II, cohesin, PRC1, and H3K27me3 (2L_empty_A, 2L_empty_B, 2R_empty_B) relative to a fourth empty site (2R_empty_A). Error bars show the standard error for all PCR replicates. The primer sequences and genomic locations of the amplicons are given below the graph. The antibodies used [27], [28] are different from those used for genome-wide mapping. (TIF) [file pgen.1003560.s005.tif]

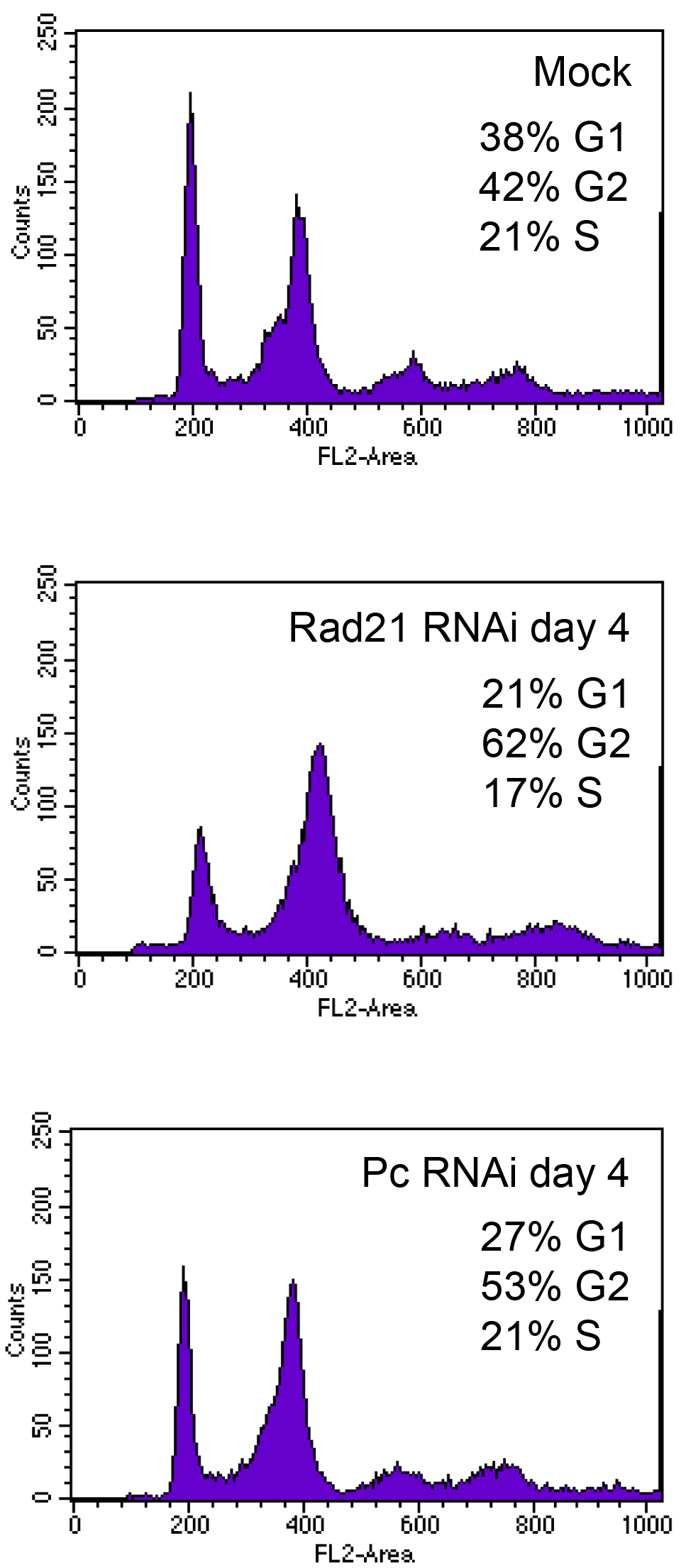

Supplement: Figure S6 — Cell cycle analysis of BG3 cells depleted for Rad21 and Pc. The panels show fluorescence-activated cell sorting analysis of mock-RNAi treated BG3 cells, and BG3 cells depleted by ∼80% for Rad21 or Pc. The estimated percentages of cells in G1, G2 and S phase are indicated. (TIF) [file pgen.1003560.s006.tif]

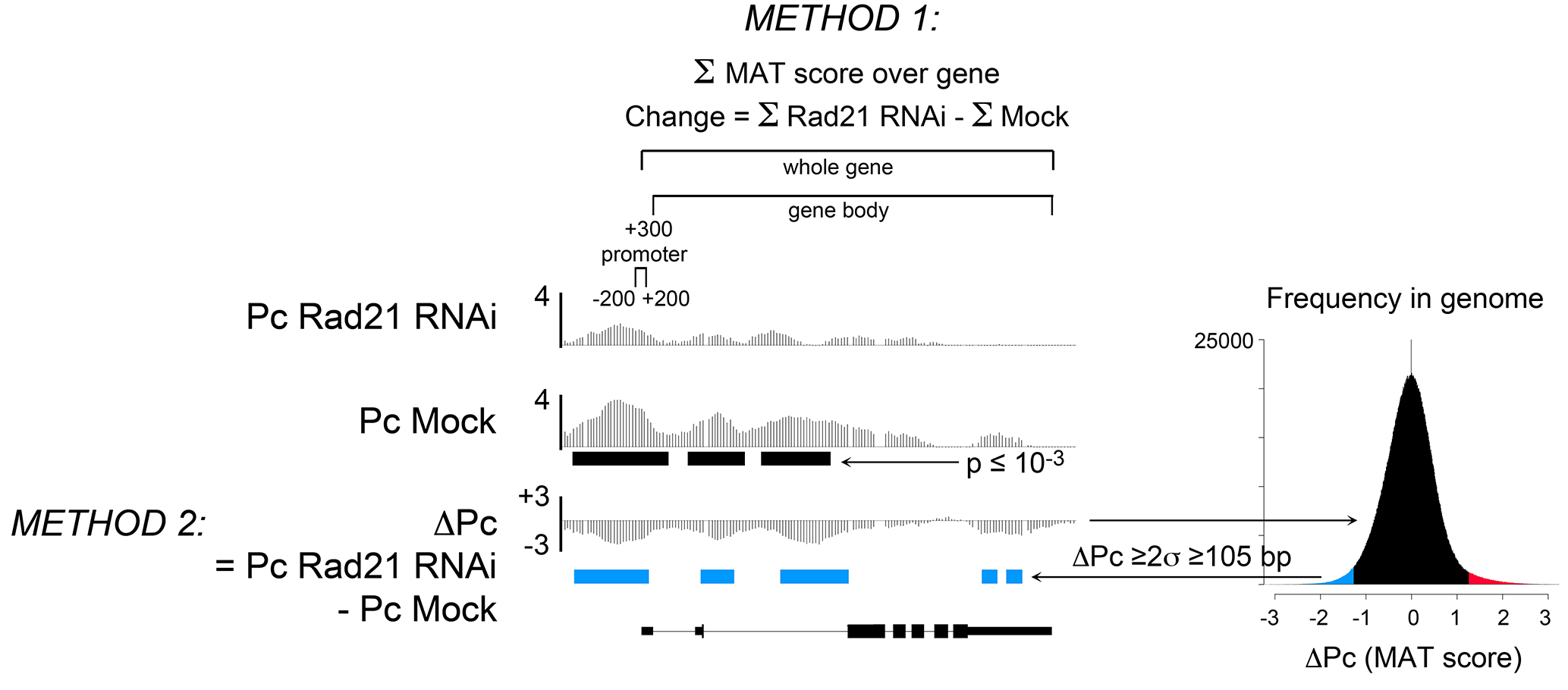

Supplement: Figure S7 — Methods used to measure changes in protein occupancy using ChIP-chip data. Both methods used the MAT scores calculated using at least two independent biological replicates for each measurement. This ChIP-chip method provides a highly quantitative and reproducible measure of binding. For instance, as shown in Figure S3, the genome-wide correlation between the Pc MAT scores in the anterior and posterior halves of the wing was 0.98, which compares two independent anterior chromatin preparations and ChIP experiments to two independent posterior chromatin preparations and ChIP experiments. In method 1, the MAT score for all microarray features contained within each annotated transcription unit, gene body, or promoter region were summed for the RNAi-depleted and mock control cells, and the total in the control for each gene was subtracted from the total in the RNAi-depleted cells to determine the total change in binding for each gene. To calculate the pause index, the median of the MAT scores in the promoter region was divided by the median in the gene body. In method 2, the MAT score for each microarray feature in the mock control cells was subtracted from the MAT score for each feature in the RNAi-depleted cells to generate a genome-wide array of Δ values for all microarray features. The distribution of these genome-wide Δ values were visualized by a histogram as shown on the right, and the mean and median values were calculated to ensure that both were close to zero, and that the distribution was close to normal. The standard deviation of this distribution was calculated (typically between 0.6 to 1 MAT unit), and then all regions in the genome in which the ΔMAT score deviated from the median Δ by at least two standard deviations for at least three microarray features in a row (typically 105 bp) were mapped to detect significant changes in binding. These regions were aligned with the genome annotation to determine which genes have a change in binding. In practice, most [file pgen.1003560.s007.tif]

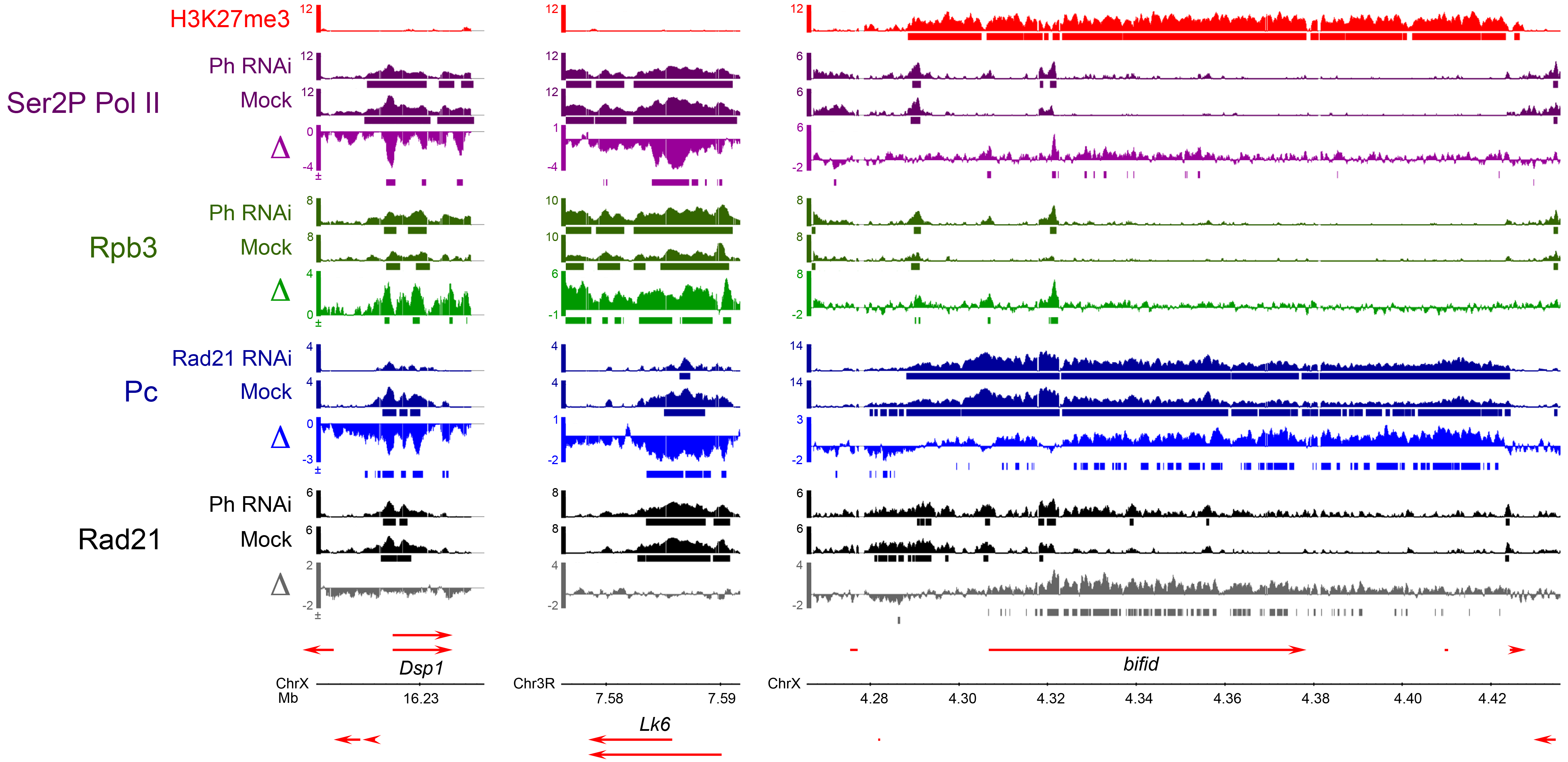

Supplement: Figure S8 — Examples of changes in Rad21 and Pol II levels upon Ph depletion, and changes in Pc binding upon Rad21 depletion at active and PcG-silenced genes. The left and middle panels show the active Dsp1 transcription factor and Lk6 protein kinase genes, and the right panel shows the PcG-silenced bifid (bi) transcription factor gene. (TIF) [file pgen.1003560.s008.tif]

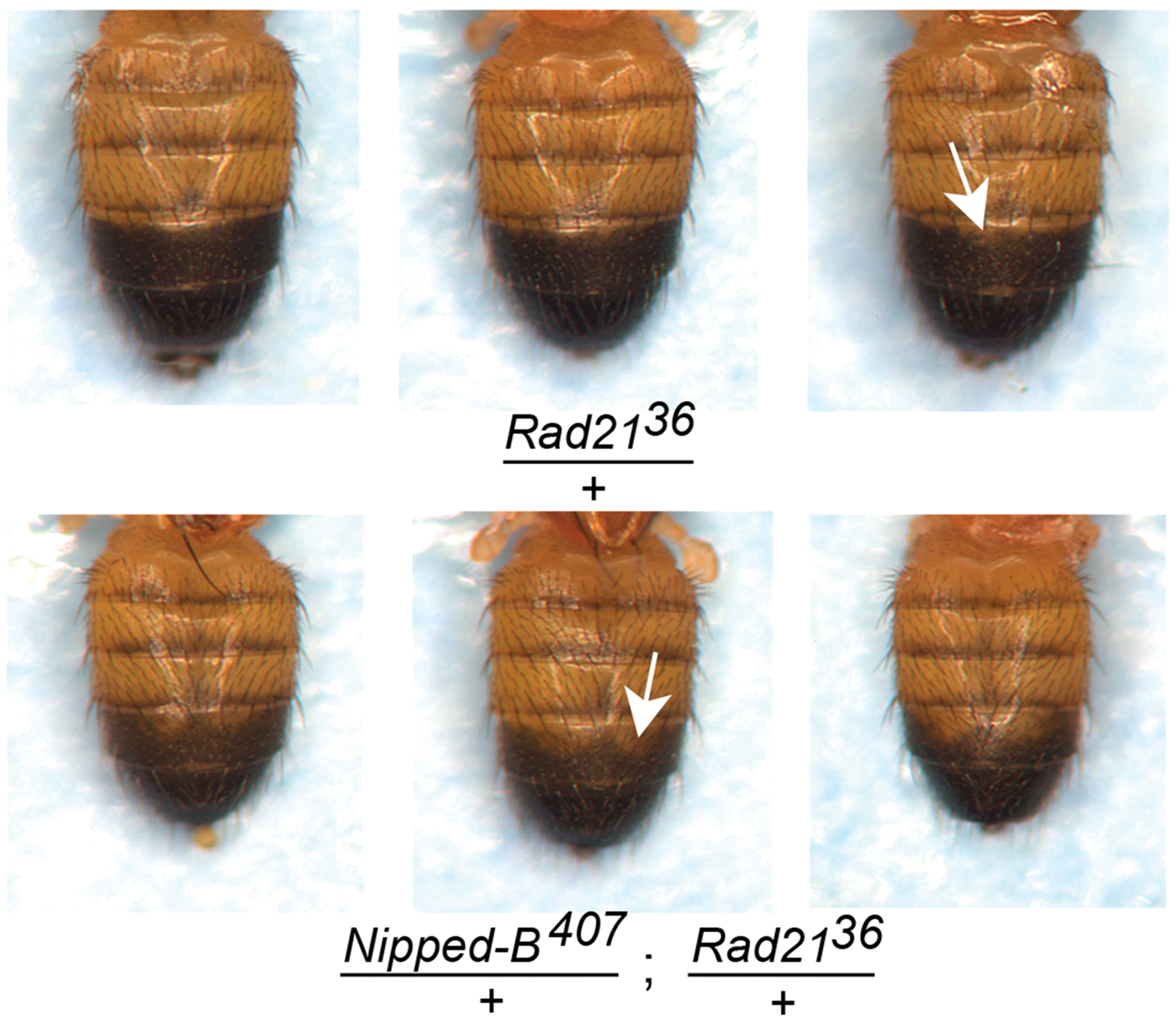

Supplement: Figure S9 — Abdominal transformations of cohesin mutants. Heterozygous Rad2136 (vtd36) exhibits mild, partially penetrant (10 to 20%) A5 to A4 transformations. Arrows indicate regions with lighter than normal pigmentation. The representative left-most and central panels on the top show Rad2136/+ pigmentation phenotype close to wild-type, while the right-most is an instance where pigmentation is significantly reduced (arrow), indicating A5 towards A4 transformation. The transformation is significantly stronger in Nipped-B407/+; Rad2136/+ trans-heterozygotes (bottom row) and penetrance is 100%. (TIF) [file pgen.1003560.s009.tif]

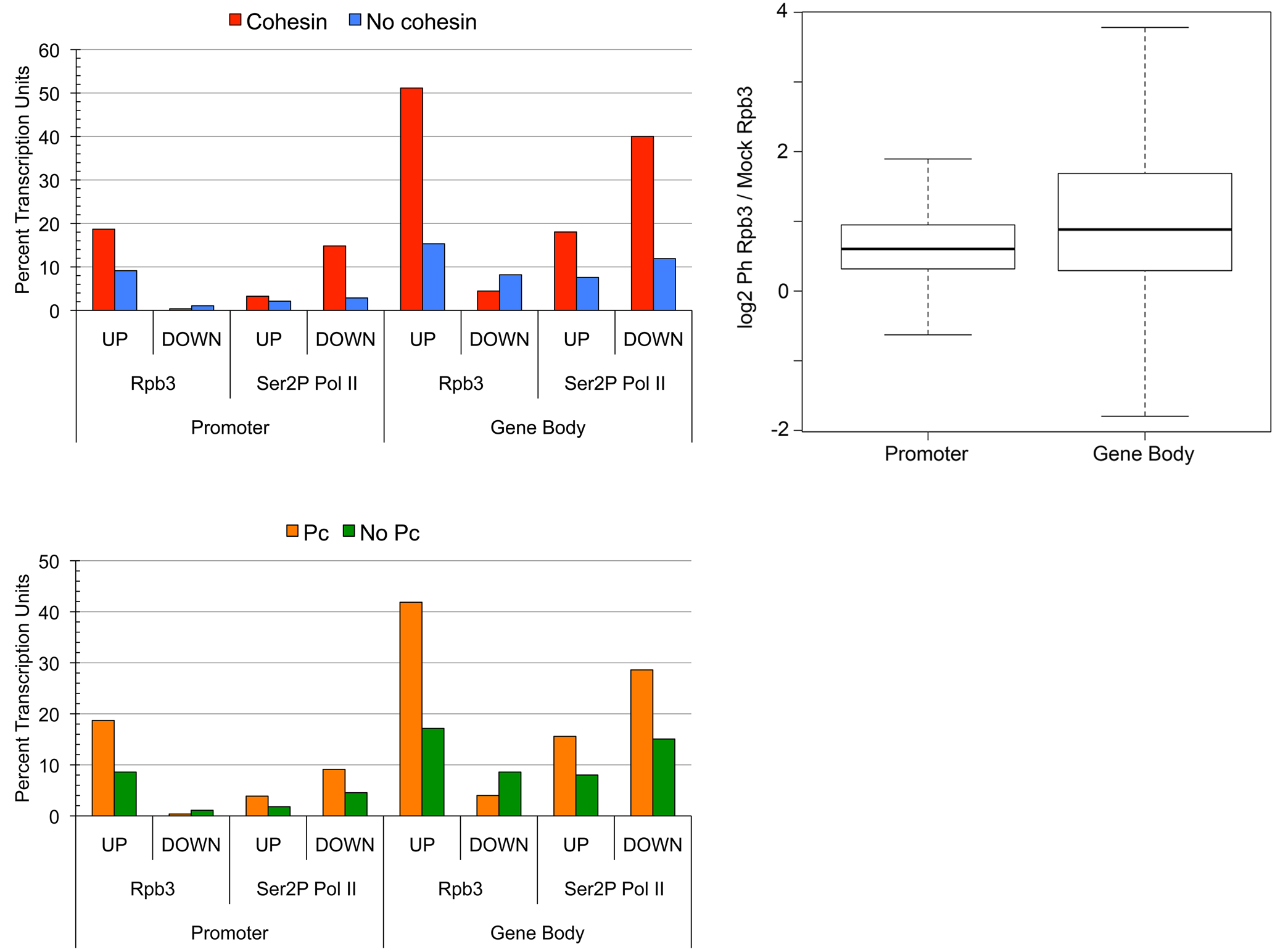

Supplement: Figure S10 — Ph depletion preferentially alters Pol II occupancy at cohesin-PRC1 binding genes in BG3 cells. The top bar graph shows the percentage of genes that bind cohesin (red) or that don't bind cohesin (blue) that show an increase (UP) or decrease (DOWN) in Pol II occupancy upon Ph depletion. Cohesin binding was determined at p≤10−3, and changes in binding were determined by method 2 in Figure S7. The promoter was defined as the 200 bp region surrounding the annotated transcription start site, and the gene body was defined as the rest of the annotated transcription unit. The bottom panel shows the same scoring for genes that bind Pc (orange) or don't bind Pc (green). Pc binding was determined at p≤10−3. The box plot in the upper right shows that the fold-changes in total Pol II (Rpb3) density in the gene body (method 1, Figure S7) are usually larger than at promoters. (TIF) [file pgen.1003560.s010.tif]
