# Supplementary material for: Cohesin and Polycomb Proteins Functionally Interact to Control Transcription at Silenced and Active Genes
Source: PLoS Genet. 2013 Jun 20;9(6):e1003560. doi: 10.1371/journal.pgen.1003560 (PMC3688520; doi:10.1371/journal.pgen.1003560)
Supplement: Table S1 — Genome-wide correlation coefficients for ChIP signals in wing imaginal discs. (DOC) [file pgen.1003560.s011.doc]

**Table S1.** **Genome-wide correlation coefficients for ChIP signals in wing imaginal discs.**

| **Comparison** | **Whole wing discs** | **Posterior wing discs** |
| --- | --- | --- |
| Rad21-Nipped-B | 0.91 | n.d. |
| Rad21-Pol II | 0.63 | n.d. |
| Rad21-H3K27me3 | 0.24 | 0.11 |
| Rad21-Pc | n.d. | 0.75 |
| Rad21-Ph | 0.82 | n.d. |

n.d. = not determined
