# Supplementary material for: Cohesin and Polycomb Proteins Functionally Interact to Control Transcription at Silenced and Active Genes
Source: PLoS Genet. 2013 Jun 20;9(6):e1003560. doi: 10.1371/journal.pgen.1003560 (PMC3688520; doi:10.1371/journal.pgen.1003560)
Supplement: Table S2 — Genome-wide data used in this study. (DOC) [file pgen.1003560.s012.doc]

**Table S2. Genome-wide data used in this study.**

| **Data** | **Cell or Tissue** | **Source** |
| --- | --- | --- |
| Rad21 ChIP | Whole 3rd instar wing disc | This study, GSE42106 |
| Rad21 ChIP | Anterior 3rd instar wing disc | This study, GSE42106 |
| Rad21 ChIP | Posterior 3rd instar wing disc | This study, GSE42106 |
| Rad21 ChIP | BG3 cells | Ref [32], GSE42399 |
| Rad21 ChIP | BG3 cells, Ph RNAi treated | This study, GSE42106 |
| Nipped-B ChIP | Whole 3rd instar wing disc | This study, GSE42106 |
| Nipped-B ChIP | BG3 cells | Ref [12], GSE9248 |
| Smc1 ChIP | BG3 cells | Ref [12], GSE9248 |
| Pol II | Whole 3rd instar wing disc | This study, GSE42106 |
| H3K27me3 ChIP | BG3 cells | Ref [26], GSE25321 |
| H3K27me3 ChIP | BG3 cells | Ref [25], GSE18100 |
| H3K27me3 ChIP | Whole 3^rd^ instar wing disc | This study, GSE42106 |
| H3K27me3 ChIP | Anterior 3rd instar wing disc | This study, GSE42106 |
| H3K27me3 ChIP | Posterior 3rd instar wing disc | This study, GSE42106 |
| Pc, Pc-RJ ChIP | Anterior 3rd instar wing disc | This study, GSE42106 |
| Pc, Pc-RJ ChIP | Posterior 3rd instar wing disc | This study, GSE42106 |
| Pc, Pc-RJ ChIP | BG3 cells | This study, GSE42106 |
| Pc, Pc-RJ ChIP | BG3 cells, Rad21 RNAi treated | This study, GSE42106 |
| Pc, Pc-VP ChIP | Whole 3rd instar wing disc | This study, GSE42106 |
| Pc, Pc-VP ChIP | BG3 cells | Ref [26], GSE25321 |
| Ph ChIP | Whole 3rd instar wing disc | This study, GSE42106 |
| Psc ChIP | Whole 3rd instar wing disc | This study, GSE42106 |
| Rpb3 ChIP | BG3 cells | Ref [32], GSE42399 |
| Rpb3 ChIP | BG3 cells, Ph RNAi treated | This study, GSE42106 |
| Ser2P Pol II ChIP | BG3 cells | Ref [32], GSE42399 |
| Ser2P Pol II ChIP | BG3 cells, Ph RNAi treated | This study, GSE42106 |
| mRNA | Whole 3rd instar wing disc | This study, GSE42106 |
| mRNA | BG3 cells | This study, GSE42106 |
| mRNA | BG3 cells, Ph RNAi treated | This study, GSE42106 |
