# Supplementary material for: Cohesin and Polycomb Proteins Functionally Interact to Control Transcription at Silenced and Active Genes
Source: PLoS Genet. 2013 Jun 20;9(6):e1003560. doi: 10.1371/journal.pgen.1003560 (PMC3688520; doi:10.1371/journal.pgen.1003560)
Supplement: Table S4 — Genetic interactions between heterozygous sister chromatin cohesion and PRC1 subunit mutations. (DOC) [file pgen.1003560.s014.doc]

**Table S4**. **Genetic interactions between heterozygous sister chromatin cohesion and PRC1 subunit mutations.**

| **Mutation** | **Phenotype** | ***vtd^6^*** | ***vtd^36^*** | ***Smc1^exc46^*** | ***Nipped-B^407^*** | ***pds5^e3^*** | ***pds5^e6^*** |
| --- | --- | --- | --- | --- | --- | --- | --- |
| *Pc^1^* | sex comb | S | s | S | S | E | o |
| *Pc^4^* | sex comb | s | S | o | S | E | E |
| *Psc^1^* | sex comb | s | s | o | o | o | o |
| *Sce^1^* | sex comb | s | s | S | o | o | E |
| *ph^401^* | sex comb | S | s | o | o | o | o |
| *ph^401^* | A4 -> A5 | S | S | o | o | o | o |

S = strong suppression; s = weak suppression; E = strong enhancement; e = weak enhancement; o = no significant effect.
